# Behavioral analysis of motor and non-motor impairment in rodent models of Parkinson's disease

**DOI:** 10.3389/fnagi.2024.1464706

**Published:** 2024-12-23

**Authors:** Razan Sheta, Morgan Bérard, Dylan Musiol, Laura Martínez-Drudis, Abid Oueslati

**Affiliations:** ^1^CHU de Québec-Université Laval Research Center, Neuroscience Axis, Québec City, QC, Canada; ^2^Department of Molecular Medicine, Faculty of Medicine, Université Laval, Québec City, QC, Canada

**Keywords:** animal models of PD, toxin-based PD model, genetic models of PD, motor impairment, cognitive performance

## Abstract

Parkinson's disease (PD) is a prevalent neurodegenerative disorder characterized by the degeneration of dopamine neurons in the substantia nigra pars compacta, leading to motor and non-motor symptoms. While motor symptoms such as rigidity, tremor, bradykinesia/akinesia, and postural instability are well-recognized, non-motor symptoms including cognitive decline, depression, and anxiety also significantly impact patients' quality of life. Preclinical research utilizing animal models has been instrumental in understanding PD pathophysiology and exploring therapeutic interventions. Various approaches, including genetic manipulations and toxin-induced insults, aim to recapitulate both motor and non-motor aspects of PD in animal models. However, no single model fully replicates the complex spectrum of PD symptoms. Behavioral assessments play a crucial role in evaluating motor impairments in PD animal models, focusing on the manifestation of Parkinsonian motor phenotype. These assessments encompass locomotor activities, motor behavior abnormalities, and induced rotational behavior. Similarly, non-motor features are assessed through tests evaluating behavioral alterations such as depression, anxiety, and cognitive impairment. Although numerous animal models of PD have been developed, including non-human primates and both mammalian and non-mammalian species, this review focuses on motor and non-motor testing methodologies in rodent models, which are the most commonly used. Emphasizing genetic and toxin-induced PD models in mice and rats, we highlight key testing strategies and the significance of each method in addressing specific research questions and interpreting experimental data. By providing a comprehensive overview of these testing approaches, this review aims to advance understanding and foster progress in PD research.

## 1 Introduction

Parkinson's disease (PD) is the second most common neurodegenerative disease, characterized by the degeneration of the dopamine (DA) neurons of the substantia nigra *pars compacta* (SNc) leading to striatal DA deficiency (Jankovic, 2008; Elbaz et al., 2016) and the accumulation of pathological α-synuclein (α-syn) (Lashuel et al., 2013; Obeso et al., 2010). Besides the neuronal loss, PD is characterized by an array of motor and non-motor symptoms. The primary motor symptoms include rigidity, tremor, bradykinesia/akinesia, and postural instability (Jankovic, 2008; Elbaz et al., 2016). Non-motor symptoms include cognitive impairments, such as deficits in attention, recognition, and working memory, often accompanied by an increase in depression and anxiety in PD patients; and these cognitive symptoms seem to appear in the earlier stages of the disease adversely affecting the quality of life of PD patients (Jankovic, 2008; Elbaz et al., 2016).

These motor and non-motor symptoms primarily stem from dysfunctions in both dopaminergic and non-dopaminergic systems within the brain. In fact, dopamine depletion in the basal ganglia leads to motor symptoms such as bradykinesia, akinesia, and muscle rigidity. In contrast, dopamine depletion in other brain regions, including the locus coeruleus, thalamus, and amygdala, is associated with non-motor symptoms like depressive symptoms (Remy et al., 2005). Furthermore, evidence highlights the role of non-dopaminergic systems in PD symptoms, particularly the serotonergic system. Clinical and post-mortem studies have shown significant reductions in serotonin markers, correlating with cognitive impairments such as depression, fatigue, and hallucinations, as well as motor deficits like tremor and levodopa-induced dyskinesia (Kish, 2003; Kish et al., 2008; Politis and Loane, 2011). Finally, acetylcholine (ACh) dysfunction has been linked to cognitive impairments in PD. Neuroimaging studies have revealed a significant reduction in acetylcholinesterase (AChE) activity in the cortex of PD patients (Shimada et al., 2009; Liu et al., 2015a). This reduction in cholinergic activity is often associated with dementia (Ruberg et al., 1986) and significantly reduced performance in cognitive tasks such as working memory and attention (Bohnen et al., 2006).

Unfortunately, PD remains incurable, and significant efforts have been made to better understand the disease and develop treatments that either modify the disease or improve its symptoms. In this context, preclinical research on PD has greatly benefited from the development of animal models that aim at evolving approaches to treat both motor and cognitive symptoms of PD. Many of the animal models employ methods that try to mimic features of PD, including the use of genetic manipulations, toxin induced insults, or combinations of the two approaches. The primary objective of using these PD animal models is to enhance their capacity to recapitulate, not only the neuropathological features of the disease, but also behavioral motor and non-motor symptoms (Cicchetti et al., 2009).

Specifically, behavioral assessment of motor impairments in PD animal models focuses on animals developing what is known as the parkinsonian-like motor phenotype (Brooks and Dunnett, 2009), and many of the tests used today include, and are not limited to, examining locomotor activities, abnormalities in motor behavior, and testing induced rotational behavior (Brooks and Dunnett, 2009). Non-motor features of PD are assessed with a vast array of tests that aim at evaluating behavioral alterations such as depression, anxiety, and cognitive impairment.

Of note, a wide range of motor and non-motor tests exists, varying depending on the animal model used for the study. Various species, including rodents, non-human primates (NHPs), and non-mammalian species, have been employed to model PD. Each model serves specific research purposes based on the complexity of the PD symptoms under investigation and comes with its own advantages and limitations.

Rodents, including mice and rats, are the most used and practical choice for evaluating various aspects of PD pathological features due to their relevance to human disease. They are cost-effective and genetically modifiable, allowing for the creation of transgenic models that overexpress α-syn or other PD-related proteins (Mor et al., 2017; Ip et al., 2017). Rodent models also mimic many key pathological and behavioral features of PD, such as selective dopaminergic neuronal degeneration and the manifestation of motor (e.g., bradykinesia, tremors) and non-motor symptoms (e.g., depression and cognitive decline) (Khan et al., 2023; Brooks and Dunnett, 2009). Additionally, rodent models are amenable to high-throughput studies, making them ideal for exploring PD-related molecular pathways (Konnova and Swanberg, 2018). However, their limitations include a simpler brain structure compared to humans. Rodents lack complex cognitive functions, and their neural circuits differ from those of humans, particularly in areas related to higher-order motor control and cognition (Nakajima and Schmitt, 2020; Xu et al., 2022). Therefore, while rodents are excellent for studying basic disease mechanisms and screening treatments, they may not fully capture the more complex aspects of human PD (Mangrulkar et al., 2024).

NHPs, including monkeys and apes, provide closer anatomical and physiological similarities to humans, offering superior translational validity in studies of motor and non-motor symptoms (Liang et al., 2023; Morissette and Di Paolo, 2018). NHPs have advanced cortical areas involved in motor planning, decision-making, and other cognitive functions critical in PD (Emborg, 2007; Blesa et al., 2018). Their ability to engage in complex tasks such as reaching and grasping, and their capacity for bipedal locomotion, allow researchers to study both motor dysfunction and more nuanced aspects of PD-like cognitive impairment and emotional regulation (Fitzsimmons et al., 2009; Liang et al., 2023). Despite these advantages, the use of NHPs is limited by ethical concerns, high costs, and the complexity of managing these animals in laboratory settings. Additionally, the technological demands for precise monitoring and manipulation (e.g., neurophysiological recordings) in NHPs are more complex than those in rodents (Zhang et al., 2012).

Finally, non-mammalian species, like *Caenorhabditis (C.) elegans* and *Drosophila melanogaster*, are invaluable for studying genetic and molecular aspects of PD due to their simplicity, short lifespans, and suitability for high-throughput screening. *C. elegans*, with their transparent bodies and simple nervous systems, allow for easy visualization of genetic changes and neuronal activity (Caldwell et al., 2020; Randi et al., 2023). They are particularly useful for genetic screening and identifying molecular pathways involved in PD. Similarly, *Drosophila* has been used to study the role of α-syn in PD pathogenesis (Xiong and Yu, 2018; Mizuno et al., 2010). α-Syn-expressing Drosophila have been reported to replicate several key features of human PD, including motor impairments (Feany and Bender, 2000), Lewy body-like inclusion formation (Karpinar et al., 2009; Auluck et al., 2002; Chen and Feany, 2005), and dopaminergic neuron degeneration (Feany and Bender, 2000). While these models are advantageous for genetic manipulation and large-scale screening, their simplicity is a significant limitation. These species lack the neuroanatomical complexity of mammalian brains, particularly in areas related to higher-order cognitive functions and are not capable of replicating the full range of PD pathology observed in humans. They are especially limited in modeling the non-motor symptoms of PD, such as cognitive decline and mood disorders, which are better observed in more complex models like rodents or NHPs (Cooper and Van Raamsdonk, 2018; Vos and Klein, 2021; He et al., 2024).

**Figure 1:**
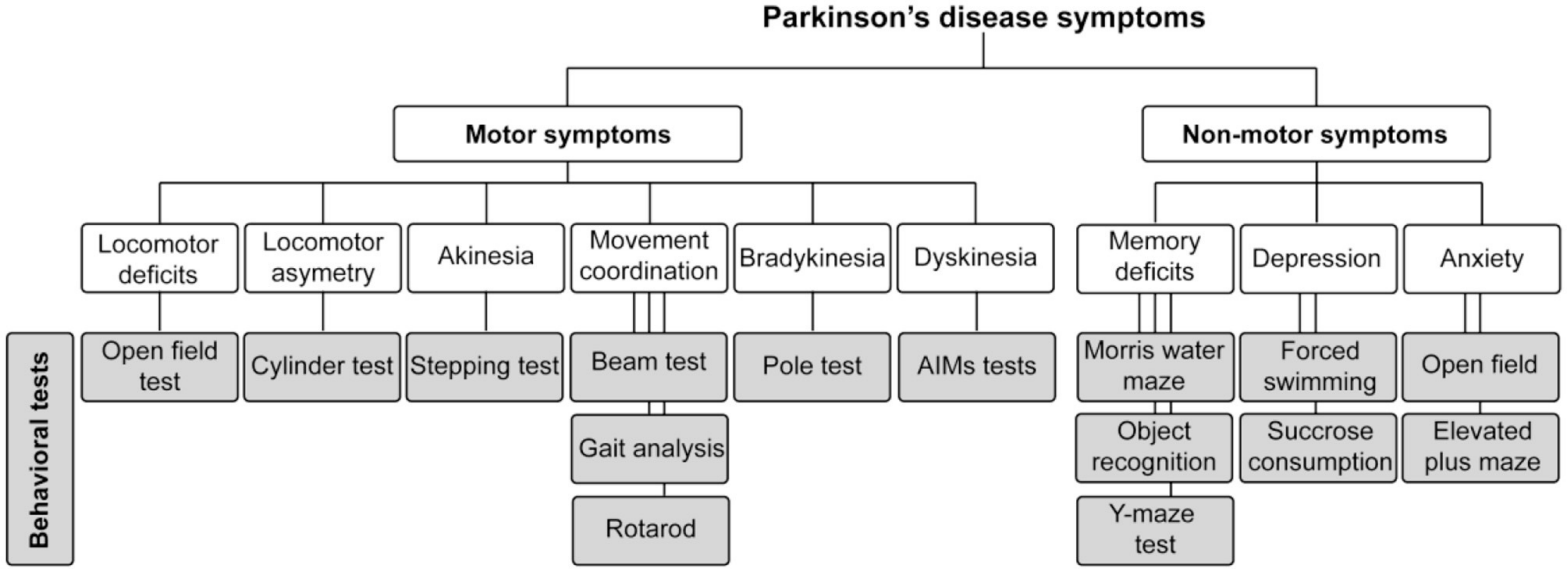
Schematic representation of motor and non-motor symptoms of Parkinson's disease, along with the behavioral tests used to evaluate these symptoms in rodent models of the disease.

Given the advantages and limitations of each animal model, it is crucial to carefully select the optimal one to ensure alignment with the specific research question and to provide clear answers. In this review, we focus on describing the motor and non-motor tests in rodent models of PD, which remain the most widely used due to their genetic malleability and cost-effectiveness (Figure 1). Specifically, we will discuss motor and non-motor tests used in both genetic and toxin-induced PD models, with a particular emphasis on the application of these experimental methods to assess PD features.

## 2 Motor behavioral tests

Motor manifestations of PD encompass bradykinesia and akinesia, marked by diminished or absent movements alongside muscle rigidity and stiffness (Schilder et al., 2017). Akinesia emerges early in PD progression, leading to gait disturbances characterized by a sensation of freezing in the limbs, postural irregularities, and challenges in executing voluntary actions like transitioning in and out of bed (Schilder et al., 2017). Various rodent models of PD have replicated akinesia symptoms through tests evaluating spontaneous or drug-induced locomotor activity. These assessments involve diverse motor tasks, including: (i) tests for spontaneous movement (such as the free movement test, cylinder test, initiation movement test, pole test, beam traversal test, gait test, and adhesive removal test), (ii) evaluation of abnormal involuntary movements (AIMs) associated with akinesia and catalepsy, and (iii) assessment of rotational behavior using the rotarod test (Figure 1).

### 2.1 Spontaneous movement tests

Spontaneous movement tests assess locomotor activity, aiding in the comprehensive evaluation of PD states in both mouse and rat models (Perlmutter, 2009). These tests are conducted to analyze various movements, enabling accurate measurement and quantification of ambulation, latency, or rearing (Seibenhener and Wooten, 2015). Several groups have made use of these movement tests in both toxin-induced and genetic models of PD.

#### 2.1.1 Open field test

**Figure 2:**
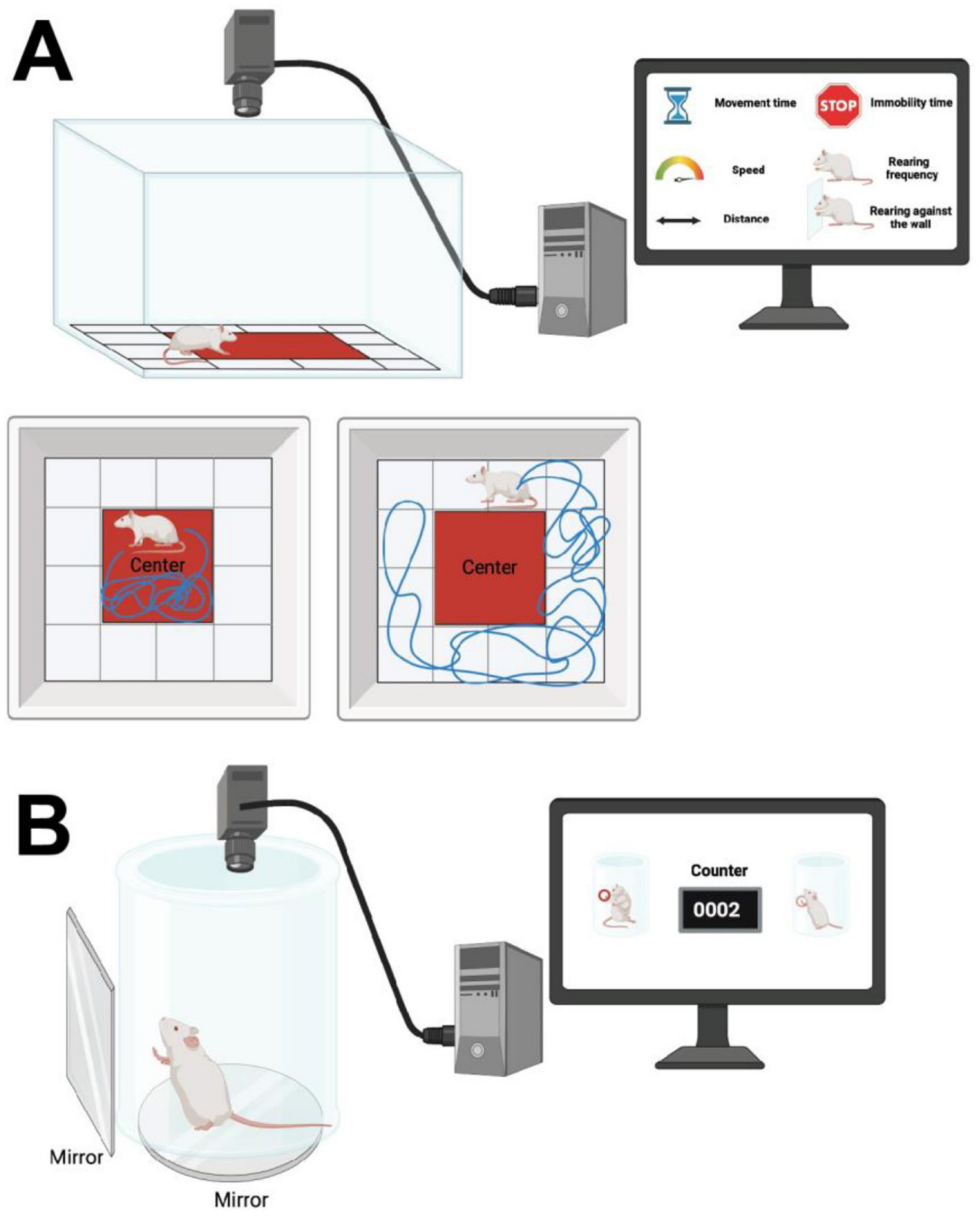
**(A)** The open field test involves a spacious cubic plexiglass enclosure, with dimensions varying based on experimental requirements. The top of the enclosure is uncovered. The field is segmented into predefined zones such as outer, middle, and inner/center. Animals are placed either in the center or one of the corners at random, allowing free exploration. Computer-based tracking programs record and analyze the animal's movements during this period. Parameters assessed include horizontal activity, movement time, movement speed (cm/s or m/s), time spent in different zones, rearing frequency (instances of standing on hind paws), rearing against the wall, hindering (sum of rearing frequency and rearing against the wall), immobility time (seconds without movement), and total distance traveled within the enclosure. **(B)** The cylinder test involves placing an animal in an open-top clear plastic cylinder with a glass mirror below to visualize movements from all directions. Forelimb activity while rearing against the cylinder wall is recorded, with scoring based on the number of forelimb contacts within 5–10 min. Variations assess weight-bearing contacts on the cylinder wall by the ipsilateral or contralateral paw relative to the lesioned hemisphere, and movements by both paws. Created using BioRender.com source.

The open field test, also known as home cage locomotion test, is a widely used method for evaluating locomotor and exploratory behaviors in experimental rodents modeling PD. This test typically involves a spacious cubic plexiglass enclosure, which can vary in dimensions depending on the experimental requirements, commonly sized at either 40 × 40 × 30 cm, 1 × 1 × 1 m, or 72 × 72 × 36 cm (Seibenhener and Wooten, 2015; Tatem et al., 2014). The top of the enclosure remains uncovered, and the open field is often segmented into predefined zones such as outer, middle, and inner/center. Animals are placed either in the center or randomly assigned to one of the corners, allowing them to freely explore for 30 min to 1 h (Seibenhener and Wooten, 2015; Tatem et al., 2014). Throughout this exploratory period, computer-based tracking programs record and analyze the animal's movements, facilitating subsequent statistical analysis (Seibenhener and Wooten, 2015; Tatem et al., 2014) (Figure 2A). Parameters assessed during the open field test include horizontal activity, movement time, movement speed (measured in cm/s or m/s), time spent in different zones, rearing frequency (instances of standing on hind paws), rearing against the wall, hindling (sum of rearing frequency and rearing against the wall), immobility time (seconds of no movement), and total distance traveled within the enclosure (Seibenhener and Wooten, 2015; Tatem et al., 2014; Santiago et al., 2010).

In the early stages of PD, rodents may show mild reductions in locomotor activity and increased anxiety-like behavior. These changes can be subtle and may require detailed analysis to detect. As PD progresses, more pronounced reductions in locomotor activity are observed. Rodents may exhibit decreased exploratory behavior and increased time spent in the periphery of the open field, indicating both motor impairment and heightened anxiety. In advanced stages, severe motor impairments become evident. Rodents may show significant reductions in movement, increased freezing behavior, and a marked decrease in exploratory activity (Seibenhener and Wooten, 2015). These changes reflect the severe motor deficits and anxiety associated with advanced PD.

Results from the test can be observed by examining multiple factors: (1) decrease in the distance traveled, in the open field can indicate motor impairments, (2) reduced velocity reflecting bradykinesia, (3) reduction in the number of rears can indicate decreased exploratory activity and motor function, (4) reduced grooming behavior can reflect both motor and non-motor symptoms of PD, such as apathy and motor deficits, (5) increased freezing episodes are indicative of motor impairments and can correlate with the severity of PD, lastly (6) an increase in the latency to move can indicate akinesia (Gould et al., 2009).

Behavioral studies utilizing the 1-methyl-4-phenyl-1,2,3,6-tetrahydropyridine (MPTP) model are less common in rats due to the lower toxicity of MPTP in this species (Dunnett and Lelos, 2010). However, studies applying MPTP have demonstrated significant effects in mouse models, including dopamine depletion upon administration (Mingazov et al., 2018) (Table 1). In the MPTP neurotoxin model, substantial and progressive decreases in locomotor activity in mouse models have been reported, as assessed by the open field test (Meredith and Rademacher, 2011; Zhang et al., 2023). Furthermore, studies have reported pronounced behavioral impairment across various parameters measured in the open field test, including locomotion distance and speed, peripheral activity, and frequency and duration of rearing (Schwarting et al., 1999; Zhang et al., 2016; Liu et al., 2015b; Essawy et al., 2017; Zhang et al., 2023). Nonetheless, there are discrepancies regarding the model's association with motor impairment, as some studies have found no significant effects of MPTP exposure on locomotor activity in animals (Colotla et al., 1990; Rousselet et al., 2003).

**Table 1:** Summary of motor tests evaluated in Parkinson's disease models.

		**Motor tests**								
		**Open field or home cage locomotion tests**	**Cylinder test**	**Stepping test**	**Beam test**	**Pole test**	**Gait analysis**	**AIMs test**	**Rotarod test**	**References**
**Neurotoxin**	6-OHDA	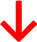 ^1^	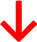 ^2^	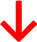 ^3^	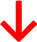 ^4^	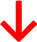 ^5^	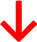 ^6^	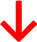 ^7^ with L-DOPA and others	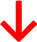 ^8^	1. Carvalho et al., 2013; Cui et al., 2024; Slezia et al., 2023 2. Schallert et al., 2000; Tillerson et al., 2001; Lundblad et al., 2004; Iancu et al., 2005; Vercammen et al., 2006; Glajch et al., 2012; Boix et al., 2015 3. Tillerson et al., 2001; Glajch et al., 2012; Boix et al., 2015Fang et al., 2006 4. Glajch et al., 2012 Mendes-Pinheiro et al., 2021 Allbutt and Henderson, 2007; Nourmohammadi et al., 2022 5. Matsuura et al., 1997; Glajch et al., 2012; Ashrafi et al., 2017 6. Boix et al., 2018; Zhou et al., 2015; Baldwin et al., 2017 7. Wan et al., 2017; Andersson et al., 1999; Cenci and Lundblad, 2007; Aristieta et al., 2012; Issy et al., 2015 Iancu et al., 2005; Putterman et al., 2007; Tronci et al., 2012. Ungerstedt, 1971; Creese et al., 1977; Waddington et al., 1979; Silverman and Ho, 1981. 8. Monville et al., 2006; Haddadi et al., 2014 Rozas et al., 1997; Rozas and Labandeira Garcia, 1997; Campos et al., 2013 1. Mingazov et al., 2018; Zhang et al., 2023; Schwarting et al., 1999; Liu et al., 2015a; Zhang et al., 2016; Essawy et al., 2017; Colotla et al., 1990; Rousselet et al., 2003. 2. Tonges et al., 2012; Fredriksson et al., 1990; Fredriksson and Archer, 1994; Fredriksson et al., 1994 3. Olsson et al., 1995 4. Quinn et al., 2007, 2008; Anandhan et al., 2010; Hong et al., 2015; Singsai et al., 2015; Rommelfanger et al., 2007; Ramirez-Carreto et al., 2023 5. Ogawa et al., 1985, 1987; Tasaki et al., 1991 6. Amende et al., 2005; Hampton and Amende, 2010; Wang et al., 2012b; Broom et al., 2017 Geldenhuys et al., 2015 7. Rommelfanger et al., 2007; Gupta et al., 1986 Lazzara et al., 2015. 8. Rozas et al., 1998; Ayton et al., 2013 1. Richter et al., 2007; Alam et al., 2004; Fleming et al., 2004b; Bassani et al., 2014; Von Wrangel et al., 2015; Sharma et al., 2016; Zou et al., 2023; Wang et al., 2024 2. Darbinyan et al., 2017b; Landau et al., 2022; Ameen et al., 2017; Darbinyan et al., 2017a 3. Mulcahy et al., 2011, 2012; Naughton et al., 2017, 2016 4. Zhou et al., 2016; Sharma et al., 2016; Ramkumar et al., 2018 5. Fujikawa et al., 2005; Zaitone et al., 2012; Liu et al., 2015b 6. Wen et al., 2011; Madiha et al., 2017 8. Magdy et al., 2022 1. Litteljohn et al., 2008; Rojo et al., 2007 2. Chinta et al., 2018; Cristovao et al., 2020 3. Bobyn et al., 2012; Richter et al., 2017 4. Campos et al., 2013. 1. Thiruchelvam et al., 2000a,b; Cicchetti et al., 2005 2. Bobela et al., 2014; Richter et al., 2017 3. Thiruchelvam et al., 2003 4. Bobyn et al., 2012; Richter et al., 2017 5. Hou et al., 2018 6. Tinakoua et al., 2015.
	MPTP	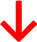 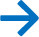 ^1^	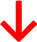 ^2^	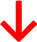 ^3^	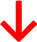 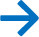 ^4^	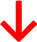 ^5^	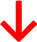 ^6^	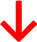 ^7^ with L-DOPA	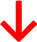 ^8^	
	Rotenone	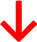 ^1^	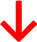 ^2^	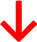 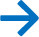 ^3^	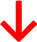 ^4^	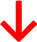 ^5^	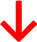 ^6^	?	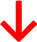 ^8^	
	Paraquat	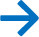 ^1^	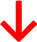 ^2^	?	?	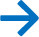 ^3^	?	?	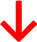 ^4^	
	Paraquat + Maneb	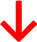 ^1^	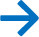 ^2^	?	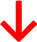 ^3^	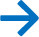 ^4^	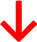 ^5^	?	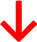 ^6^	
**Genetic model**	Thy1 α-syn	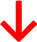 ^1^	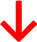 ^2^	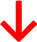 ^3^	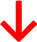 ^4^	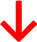 ^5^	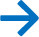 ^6^	?	?	1. Song et al., 2015 2. Chesselet et al., 2012; Oliveras-Salva et al., 2013 3. Chesselet et al., 2012; Ulusoy et al., 2010; Decressac et al., 2012; Phan et al., 2017. 4. .Fleming et al., 2004a 5. Fleming et al., 2004a; Chesselet et al., 2012 6. Fleming et al., 2004a, 2006. 1. Unger et al., 2006; Paumier et al., 2013 2. Koprich et al., 2011 3. Lelan et al., 2011; Bido et al., 2017 4. Kim et al., 2018 5. Gispert et al., 2003; Hamill et al., 2012; Lin et al., 2012; Paumier et al., 2013; Tatenhorst et al., 2016 6. Gispert et al., 2003; Paumier et al., 2013; Graham and Sidhu, 2010; Oaks et al., 2013 1. Gomez-Isla et al., 2003; Yavich et al., 2005; Oksman et al., 2009; Yin et al., 2023 2. Oksman et al., 2009; Gaugler et al., 2012 3. Lelan et al., 2011. 4. Plaas et al., 2008; Ekmark-Lewen et al., 2018 5. Plaas et al., 2008; Mendritzki et al., 2010 6. Plaas et al., 2008; Yan et al., 2017 1. Lu et al., 2009 2. Goldberg et al., 2003; Perez and Palmiter, 2005 3. Perez and Palmiter, 2005; Pinto et al., 2018 4. Perez and Palmiter, 2005 5. Goldberg et al., 2003; Zhu et al., 2007 1. Lu et al., 2009 2. Kelm-Nelson et al., 2018a,b 3. Dave et al., 2014 4. Gispert et al., 2009; Kelm-Nelson et al., 2018a 5. Glasl et al., 2012. 6. Zhou et al., 2007; Gispert et al., 2009 1. Chen et al., 2005 2. Perez and Palmiter, 2005 3. Kim et al., 2005; Chandran et al., 2008 4. Chandran et al., 2008 5. Chen et al., 2005; Dave et al., 2014, 1. Hinkle et al., 2012; Bichler et al., 2013; 2. Bichler et al., 2013 Lee et al., 2015 3. Pischedda et al., 2021 4. Adeosun et al., 2017; Xiong et al., 2017 5. Seegobin et al., 2020 6. Bichler et al., 2013
	α-syn A53T	Hyperactivity^1^	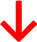 ^2^	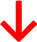 ^3^	?	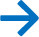 ^4^	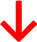 ^5^	?	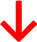 ^6^	
	α-syn A30P	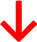 ^1^	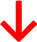 ^2^	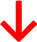 ^3^	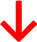 ^4^	?	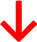 ^5^	?	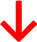 ^6^	
	Parkin	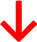 ^1^	?	?	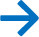 ^2^	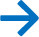 ^3^	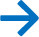 ^4^	?	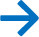 ^5^	
	Pink1	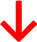 ^1^	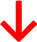 ^2^	?	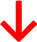 ^3^	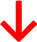 ^4^	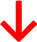 ^5^	?	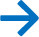 ^6^	
	DJ-1	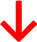 ^1^	?	?	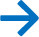 ^2^	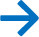 ^3^	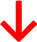 ^4^	?	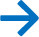 ^5^	
	LRRK2	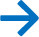 ^1^	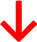 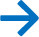 ^2^	?	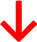 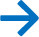 ^3^	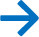 ^4^	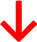 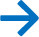 ^5^	?	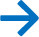 ^6^	

Motor tests: 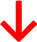
= impairment, 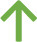
= improvement, 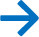
=no significant change or no effect. ? = no reported studies.

Spontaneous movement tests have also been employed in other extensively studied neurotoxin models of PD. Exposure to rotenone leads to dopamine cell loss, resulting in PD-like motor symptoms such as bradykinesia, stiffness, rigidity, slowness of movement, and postural instability (Johnson and Bobrovskaya, 2015; Richter et al., 2007). Numerous studies have reported motor abnormalities following prolonged rotenone exposure, confirmed through open field behavioral analysis in both mice and rats (Fleming et al., 2004b; Von Wrangel et al., 2015; Alam et al., 2004; Sharma et al., 2016; Bassani et al., 2014; Zou et al., 2023; Wang et al., 2024) (Table 1). These parkinsonian features were also observed in rats subjected to systemic rotenone exposure via subcutaneous osmotic pumps, where open field behavior analysis indicated significant reductions in motor activities and muscular rigidity with evident flexed postures (Sherer et al., 2003).

Similarly, administration of the neurotoxin 6-hydroxydopamine (6-OHDA) has been shown to induce parkinsonian motor features in animals, characterized by complete loss of the dopaminergic neurons, akin to MPTP and rotenone (Ungerstedt, 1968; Chia et al., 2020; Zeng et al., 2018). Studies assessing animal motor activity in the open field revealed effects of 6-OHDA lesions on total distance traveled by the animals, accompanied by a significant decrease in motor activity, as well as notable reductions in movement initiation in rats exposed to 6-OHDA (Carvalho et al., 2013; Cui et al., 2024) (Table 1). Another group examining the behavioral alterations in a 6-OHDA mouse model showed results from open field test reporting that mild motor impairments are rapidly detectable 1 week after a single low dose of striatal 6-OHDA injection, with motor impairments mostly manifesting in general horizontal locomotion and initiation of explorative locomotion, rather than motor coordination (Slezia et al., 2023).

Paraquat, another environmental toxin, is linked to the development of PD-like pathology via the degeneration of dopaminergic neurons (Andersen, 2003; Tanner et al., 2011; Cicchetti et al., 2005). However, analysis of the motor impairment after paraquat administration have not been extensively explored through a wide range of behavioral tests. Several studies failed to report significant effects or direct implications of paraquat administration on PD motor deficits. For instance, one study found no significant results regarding paraquat exposure's impact on total traveling distance or the speed of mice in the open field setting (Litteljohn et al., 2008). Similarly, another study suggested that the observed hypokinesia in mice or rats following paraquat exposure was not correlated with changes in dopamine levels, suggesting that the motor deficits observed in the open field test may not be directly linked to nigrostriatal damage but rather to systemic poisoning (Rojo et al., 2007) (Table 1).

Toxicological studies shed light on the significance of exposing animals to multiple toxic chemicals, such as paraquat and maneb, a fungicide also associated with PD pathogenesis (Desplats et al., 2012). Data from studies involving PD mice intraperitoneally injected with either paraquat alone, maneb alone, or in combination, indicated that only combined exposures to both chemicals led to a significant and progressive decrease in motor activity, as assessed by the open filed test using automated locomotor activity chambers measuring total horizontal locomotor activity by counts of photobeam breaks (Thiruchelvam et al., 2000a,b; Li et al., 2005) (Table 1). Similar results were replicated in several other studies, confirming motor deficits through behavioral assessments in the open field, indicating postural deficits, decreased speed of overall movement, and mobility in rats, as well as in mice exposed to the combined treatment of paraquat and maneb (Cicchetti et al., 2005; Thiruchelvam et al., 2002, 2003).

With the increasing number of α-syn transgenic animal models developed, numerous behavioral alterations have been documented. Locomotor deficits were reported in α-syn A30P transgenic mice, as observed in the open field and spontaneous motor tests (Gomez-Isla et al., 2003). Reports indicated significant motor impairments in mice homozygous and heterozygous for the A30P transgene, displaying rapid and progressive asymmetric motor impairment, rigidity, dystonic posturing, and loss of voluntary movements (Gomez-Isla et al., 2003). Similarly, several studies reported a significant decrease in locomotor activities in A30P transgenic mice, along with impaired motor coordination, as evaluated by the time spent in locomotion, rearing, and stereotypic movements (Yavich et al., 2005; Oksman et al., 2009; Yin et al., 2023). Motor activities were also measured in A53T transgenic mice, with studies reporting hyperactive motor activities (Unger et al., 2006; Bourdenx et al., 2015). Similar hyperactive motor activities were documented in another study, employing open field tests to distinguish activities recorded within outer and inner zones (Paumier et al., 2013). Data from this study confirmed a hyperactive phenotype accompanied by significant increases in the distance traveled in both horizontal and vertical directions (Paumier et al., 2013). Open field tests were also conducted in wild-type α-syn transgenic animal models, revealing significant reductions in motor activities at 8 and 12 weeks (Song et al., 2015). Furthermore, similar analysis were observed in transgenic models of PD under different promoters, with reports of motor impairment and behavioral dysfunction in (Thy-1)-h[A30P] α-syn mice and (Thy-1)-h[A53T] α-syn mice (Ekmark-Lewen et al., 2018) (Table 1).

Moreover, there have been limited reports on motor assessments in genetic models of PD using autosomal recessive genes including Parkin, PTEN-induced kinase 1 (PINK1), leucine-rich repeat kinase 2 (LRRK2), and deglycase (DJ-1). Nonetheless, a study examining parkin-deficient mice showed that animals can exhibit multiple late onsets associated with significant hypokinetic motor deficits when assessed using the open field test (Lu et al., 2009). Other studies also reported hypoactivity of DJ-1 deficient mice, with a decrease in spontaneous activities in the open field (Goldberg et al., 2005; Chen et al., 2005). Furthermore, studies on LRRK2 deficient mice showed no significant differences in animal behavior compared to the control using the open field test (Hinkle et al., 2012; Palomo-Garo et al., 2016; Bichler et al., 2013) (Table 1).

#### 2.1.2 The cylinder test

Locomotor asymmetry represents a key motor phenotype of PD, characterized by the initial onset of symptoms affecting one side of the body, which persists as the most prominently affected side throughout the course of the disease, even as the other side becomes affected (Djaldetti et al., 2006). In rodent models, locomotor asymmetry, sensorimotor coordination, and forelimb use are evaluated using the cylinder test (Tonges et al., 2012). This test primarily detects motor coordination differences in unilateral lesion models but also provides insights into overall rodent body coordination (Tonges et al., 2012).

Specifically, in the early stages of PD, rodents exhibit mild asymmetry in forelimb use, which progresses to increased reliance on the non-affected forelimb in the middle stages, and eventually leads to significant asymmetry in the advanced stages, with the affected forelimb rarely being used (Magno et al., 2019).

The cylinder test involves placing an animal in an open-topped clear plastic cylinder, with a glass mirror positioned below to visualize movements from underneath and all directions (Fleming et al., 2013). Behavioral assessments focus on the animal's forelimb activity while rearing against the cylinder wall, with movements recorded accordingly (Fleming et al., 2013). Scoring is based on the number of forelimb contacts (between 20 and 30 contacts) recorded within an assigned period of 5–10 min (Fleming et al., 2013) (Figure 2B). More complex variations of the test assess weight-bearing contacts on the cylinder wall of the ipsilateral or contralateral paw relative to the lesioned hemisphere, in addition to movements made by both paws (Glajch et al., 2012).

The cylinder test has been utilized in both neurotoxin and transgenic models of PD. For instance, animals lesioned with MPTP showed a significant reduction in motor coordination performance compared to control groups, as evidenced by an increased ratio of wall touches with both paws and a decrease in free rears (Tonges et al., 2012). These observations aligned with previous findings indicating reductions in motor activity, including locomotion, rearing, and total activity, in mice exposed to varying doses of MPTP (Fredriksson and Archer, 1994; Fredriksson et al., 1994, 1990) (Table 1). Similarly, the test has been applied to examine rotenone effects on motor behavior in rats, revealing deficits in behavioral features such as the number of rears over a period of weeks, confirming postural instability (Darbinyan et al., 2017b; Landau et al., 2022). Consistent findings of reduced rearing behavior in rotenone-treated animals have been reported in several other studies (Anusha et al., 2017; Morais et al., 2012; Darbinyan et al., 2017a; Ameen et al., 2017) (Table 1).

The cylinder test has proven effective in indicating forelimb motor impairment in mice and rats injected with 6-OHDA, particularly in assessing unilateral impairments (Glajch et al., 2012; Schallert et al., 2000; Tillerson et al., 2001; Lundblad et al., 2004; Iancu et al., 2005; Boix et al., 2015; Vercammen et al., 2006; Slezia et al., 2023). Moreover, rearing behavior in mice exposed to the environmental neurotoxin paraquat was analyzed using the cylinder test, revealing a decrease in motor neuron function (Chinta et al., 2018; Cristovao et al., 2020). However, studies examining the combined treatment of paraquat and maneb did not report significant effects on forelimb and hindlimb motor behavior (Richter et al., 2017; Bobela et al., 2014) (Table 1).

In PD genetic models, motor impairments detected through the cylinder test have primarily been associated with animals at advanced ages. For instance, many of the motor impairments reported using the cylinder test have been linked to animals at advanced ages, and this is true for all wild type (WT) (Gispert et al., 2003; Chesselet et al., 2012; Oliveras-Salva et al., 2013), A53T (Koprich et al., 2011; Bourdenx et al., 2015; Gispert et al., 2003) and A30P α-syn animals (Gaugler et al., 2012; Oksman et al., 2009). However, locomotor asymmetry analysis using the cylinder test has not proven highly sensitive in discriminating animals with genetic mutations such as Parkin, PINK1, LRRK2, and DJ-1 from WT controls as reviewed in Terzioglu and Galter (2008). Recent studies have shown progress in using the cylinder test to examine locomotor abnormalities in these genetic models. For example, PINK1-deficient mice displayed significant impairment in limb motor skills compared to WT controls (Kelm-Nelson et al., 2018a), while similar observations were not reported in a PINK1-deficient rat model (Kelm-Nelson et al., 2018b). Conversely, LRRK2 transgenic mice did not exhibit significant differences in rearing behavior compared to controls, with only subtle motor deficits reported (Bichler et al., 2013). At 8 months of age, a study did not reveal substantial evidence of distinct motor deficits in rats. However, by the time the rats reached 12 months of age, data indicated noteworthy increases in rearing behavior among LRRK2 transgenic rats compared to their WT controls, suggesting modest evidence of behavioral alterations overall (Lee et al., 2015). Moreover, in Parkin deficient rodent models, no significant behavioral deficits were observed at either early or later ages (Dave et al., 2014).

#### 2.1.3 Movement initiation test (stepping test)

**Figure 3:**
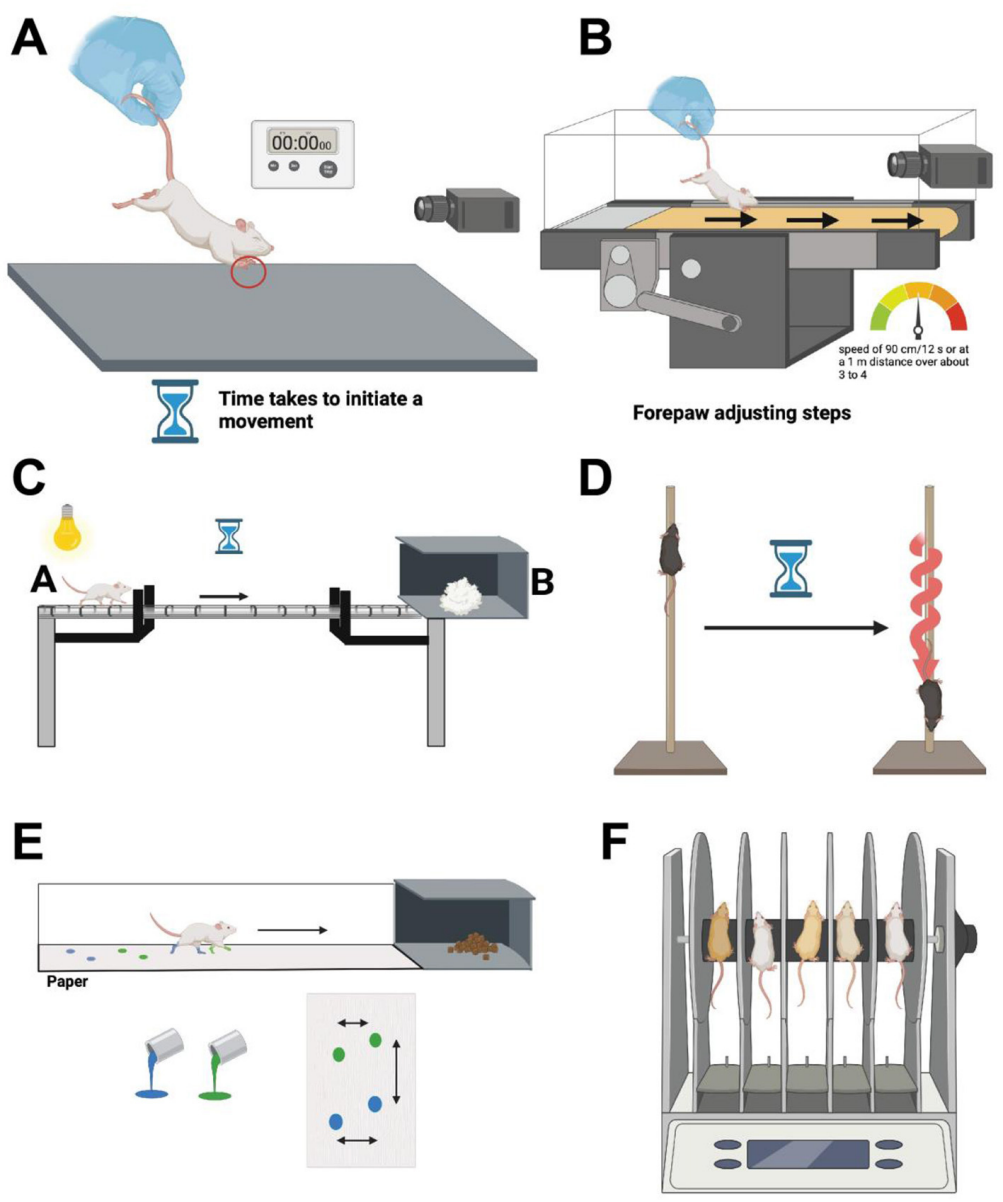
**(A)** The Movement Initiation Test involves placing an animal on a flat surface and gently lifting its hind limbs, leaving the forelimbs in contact with the ground. The animal is then moved laterally, and the number of steps taken by the forelimbs to maintain balance and posture is recorded. This test assesses the animal's ability to initiate and maintain movement, with particular focus on forelimb stepping frequency and coordination. **(B)** In the other type of forepaw test, animals are held from the rear, but in this type of test the animal is left on a stationary position to examine their forepaw movement initiation when placed on a treadmill moving at a specified rate. **(C)** The beam test involves placing a beam between a starting point **(A)** and either the animal's home cage or a darkened cage **(B)**. Observations include the time taken to traverse from A to B and the number of foot slips. The hindlimb slip ratio is calculated as (number of slips/number of total steps), with ratios for both hindlimbs averaged into one score. **(D)** In the pole test, animals are placed head-up at the top of a vertical wooden pole positioned above their cage. The test measures the time taken for the animal to turn downwards and the total time to descend the pole to the cage. **(E)** The manual gait analysis technique, or the footprint test examines limb movement. The analysis is conducted using paint colors and paper, with animals trained to walk on a narrow walkway leading to their home cages. Stride length is determined by measuring the distance between footprints, considering both forelimb and hind paw strides. **(F)** The rotarod test makes used of a rotarod apparatus, comprising a circular rod typically 3–7 cm in width, set to rotate at either constant or increasing speeds. The rodent is positioned on the rotating rod, tasked with maintaining balance to prevent falling onto a platform located approximately 30 cm below the rod. The latency to fall is recorded as the primary endpoint measure. Created using BioRender.com source.

Deficits in forepaw movements have been shown to effectively model parkinsonian akinesia, and the type of test commonly used to examine this phenomenon is referred to as the stepping test. As the disease advances to the middle stages, there is a noticeable reduction in the number of steps, reflecting increasing difficulty with gait and mobility. In the advanced stages, severe akinesia often develops, where individuals struggle to initiate movements, resulting in minimal or no steps being taken (Sirajo et al., 2022). This test is mainly based on holding the rodent by the base of the tail while keeping their hindlimbs suspended, and in turn the animal would be bearing weight on their forelimbs (Chang et al., 1999) (Figure 3). The time the animal takes to initiate a movement is then monitored for each forelimb alone or for both limbs together, initiation times for both forelimbs is then often averaged to make one score (Chang et al., 1999; Glajch et al., 2012).

There are a variety of parameters used to study movement initiation in rodent models of PD, mainly based on the type of lesion induced in the model being studied (Chang et al., 1999; Olsson et al., 1995). One such example is the application of the test referred to as forepaw adjusting steps, where rodents are moved across a table at a speed of 90 cm/12 s or at a 1 m distance over about 3 to 4 s (Chang et al., 1999; Glajch et al., 2012). Animals are usually suspended or held at the rear part with their hindlimbs lifted, while one forepaw is left held up to better concentrate bearing the weight on the other forepaw (Olsson et al., 1995) (Figure 3A). The number of adjusting steps of the non-lifted or weight-bearing forepaw is then counted over the specified time interval, the principle of this test has been very well described by Olsson et al. (1995). Another type of forepaw test is adjusted for striatal lesions, where animals are held from the rear, but in this type of test the animal is left on a stationary position to examine their forepaw movement initiation when placed on a treadmill moving at a specified rate (Chang et al., 1999) (Figure 3B). The tests can be performed while alternating between each of the two forepaws. In all types of the stepping test, the average of the several trials for each forepaw is used for analysis (Chang et al., 1999; Glajch et al., 2012; Olsson et al., 1995).

Interestingly, the stepping test has been first adapted from experiments performed on MPTP treated rats and mice (Chang et al., 1999; Olsson et al., 1995; Schallert et al., 1979; Blume et al., 2009). Reported data consistently showed a clear decline in the number of adjusting steps in MPTP treated animals (Table 1), and this decline has been suggested to directly measure the underlying motor deficit, similar to the limb akinesia observed in PD patients (Chang et al., 1999; Olsson et al., 1995; Schallert et al., 1979; Blume et al., 2009). Similarly, the stepping test has also been a useful test in examining unilateral 6-OHDA treatment in mouse models of PD, demonstrating limb-use asymmetry and other sensorimotor impairments in the 6-OHDA mouse model (Table 1) (Glajch et al., 2012; Tillerson et al., 2001; Boix et al., 2015). This test has received some critical cut-off requirements for proper assessments of stepping deficits in rodent models such as those tested in unilateral striatal lesions of 6-OHDA in rats. It has been suggested that the stepping test is not sensitive enough to distinguish the severity of 6-OHDA striatal lesions (Fang et al., 2006; Lundblad et al., 2002) despite previous claims that it is a sensitive measure of bradykinesia in unilateral 6-OHDA lesioned rats (Lindner et al., 1997). Forelimb motor dysfunctions using the stepping test have also confirmed significant motor dysfunction in rotenone-treated rats (Mulcahy et al., 2012, 2011; Naughton et al., 2017) and mice (Zhou et al., 2016), while some studies also seem to report absence of motor dysfunctions induced by rotenone in rat models (Naughton et al., 2016) (Table 1). Furthermore, motor studies using the stepping test have also been applied in transgenic models of PD, and data were indicative of a significant decrease in the performance of older α-syn rat models in the stepping test (Decressac et al., 2012; Phan et al., 2017; Ulusoy et al., 2010). Similar behavioral deficits were also observed for older A53T α-syn (Bido et al., 2017; Lelan et al., 2011) and A30P-α-syn rat models (Lelan et al., 2011). While no stepping tests have been reported for the other PD-causing genes, including parkin, DJ-1, LRRK2 and PINK1 (Table 1).

#### 2.1.4 Beam test

Several types of beam tests have been adapted to examine hind limb coordination in rodent models, providing a means to assess motor coordination in animal models of Parkinson's disease (PD). In the early stages of PD, rodents show a slight increase in traversal time. As the disease progresses to the middle stages, they experience frequent slips and longer traversal times. In the advanced stages, rodents often become unable to traverse the beam without falling (Kucinski et al., 2013).

All beam tests are based on first training the animals to cross or navigate across an elevated or inclined beam of varying lengths and narrowness, while some beam tests are based on the use of a tapered/ledged beam which allows for foot faults without the animal falling (Fleming et al., 2013; Jover et al., 2006). The beam is usually placed between a starting point (point A) and either their cage or a darkened cage (point B) (Fleming et al., 2013) (Figure 3C). Test observations are based on several criteria which include the amount of time it takes the animal to go from point A to point B, in addition to observing the number of foot slips the animals make during the traversal time (Fleming et al., 2013). The slip ratio of the hindlimb is calculated by: (number of slips/number of total steps), and the slip ratio is also often recorded for both hindlimbs and averaged to create one score, and the test is also videotaped for evaluation (Fleming et al., 2013).

Sensorimotor integration in neurotoxin-induced models of PD have been evaluated using the challenging beam walk test. In rotenone PD models, data recording showed that the time taken by rotenone-exposed mice is significantly longer on the beam traversal test (Zhou et al., 2016), similar data were observed in rat models of PD with rats showing a prominent loss of motor coordination in the beam walking test confirming PD-related motor deficits caused by rotenone (Darbinyan et al., 2017a; Ramkumar et al., 2018; Sharma et al., 2016) (Table 1). Moreover, estimation of motor coordination has been tested in MPTP mice, with several studies showing a significant increase in the beam-crossing duration of mice, which was associated with a reduction in the mice's hanging time (Hong et al., 2015; Anandhan et al., 2010; Quinn et al., 2007, 2008; Singsai et al., 2015). Conflicting results of MPTP mice models and motor deficits have also been explored in other studies, with few reports showing an absence of motor impairment in MPTP-treated mice when performing various motor tests including the beam traversal test (Rommelfanger et al., 2007; Ramirez-Carreto et al., 2023). Similar motor impairments were observed in 6-OHDA-lesioned mice using the beam test, with data showing a significant increase in the number of errors per step, the total number of steps, and the time it took to traverse the beam (Glajch et al., 2012) (Mendes-Pinheiro et al., 2021). Data observed in mice were in concordance with studies performed on rats. Parkinsonian rats demonstrated a significant increase in the initiation of the beam walking task and the total time to cross the beam (Allbutt and Henderson, 2007; Nourmohammadi et al., 2022). The paraquat + maneb model also induced reductions in motor coordination as observed using the beam test (Thiruchelvam et al., 2003) (Table 1).

In addition, the neurotoxin induction and their effects on the duration time for rodents to traverse the beam also concords with published studies examining transgenic mouse models of PD. Studies showed significantly slower rates for animals to traverse a narrow or raised beam, this was true for all mouse models overexpressing WT human α-syn (Fleming et al., 2004a), the thymus cell antigen 1 (Thy-1)-h[A30P] α-syn transgenic mouse model (Ekmark-Lewen et al., 2018; Plaas et al., 2008). While no effects on beam walking traversal time were recorded for parkin-deficient mice, and DJ-1 deficient rats (Goldberg et al., 2003; Perez and Palmiter, 2005). Significant dysfunctions of motor coordination in Pink1-deficient rats were observed (Dave et al., 2014). More recently, studies have begun to use beam tests to study the LRRK2 model of PD. However, the findings have been controversial, with some studies suggesting a decrease in motor coordination (Pischedda et al., 2021), while others have shown no changes (Skiteva et al., 2022) (Table 1).

#### 2.1.5 Pole test

Another highly sensitive test aimed at examining bradykinesia, nigrostriatal dysfunction and motor coordination in PD mice is known as the pole test (Matsuura et al., 1997). During the early stages, mice demonstrate a slight increase in the time it takes to descend the pole, reflecting mild motor impairment. In the middle stages, there are noticeable delays and hesitations as the motor deficits become more pronounced. In advanced stages, significant delays, or a complete inability to descend the pole are observed, indicating severe motor dysfunction (Matsuura et al., 1997).

In the pole test, animals are placed head-up at the top of a wooden pole positioned vertically from a designated base, that is usually the animal's cage (Matsuura et al., 1997) (Figure 3D). Animals are trained to orient themselves in descending downwards on the pole toward their cages. The test evaluates the time it takes the animal to turn downwards, in addition to the total time it needs to go down the pole to the cage from the time it is placed on the pole (Matsuura et al., 1997).

Behavioral deficits on the pole test have been examined in MPTP treated mice, confirming induced bradykinesia in these animals (Ogawa et al., 1985, 1987; Tasaki et al., 1991). Similarly, the pole test has been widely used in assessing for bradykinesia and motor coordination in 6-OHDA treated mice (Glajch et al., 2012; Matsuura et al., 1997; Ashrafi et al., 2017), with data showing 6-OHDA-lesioned mice having a significant increase in the time to turn and orient downwards, in addition to a significant increase in the total time to traverse the pole; all suggestive of behavior related disorders (Glajch et al., 2012). Studies on rotenone treatment in rats also showed significant motor performance dysfunctions when performing the pole test (Zaitone et al., 2012; Fujikawa et al., 2005), similar observations were also recently confirmed in mouse models of PD subjected to rotenone treatment (Liu et al., 2015c). Several studies reported no effects of the pesticides paraquat, or the combination of paraquat and maneb exposures, when applying the pole test confirming the absence of coordination deficits (Bobyn et al., 2012; Richter et al., 2017) (Table 1).

Transgenic mouse models such as the mouse model over-expressing full-length, WT α-syn show significant deficits in motor tests, such as deficits in orientation and coordination in the pole test (Fleming et al., 2004a; Chesselet et al., 2012), while such motor deficits were not reproducible in A53T or A30P α-syn mouse models of PD (Kim et al., 2018). Moreover, the pole test was also used to detect defects in motor coordination for other PD mouse models, and studies consistently confirmed absence of detectable motor deficits in PD models such as the Parkin (Perez and Palmiter, 2005; Pinto et al., 2018), LRRK2 (Xiong et al., 2017; Adeosun et al., 2017), and DJ-1 (Kim et al., 2005; Chandran et al., 2008). While other studies confirmed motor and traversal deficits on the pole test in PINK1 PD model (Kelm-Nelson et al., 2018a; Gispert et al., 2009) (Table 1).

#### 2.1.6 Gait analysis

The gait tests have been used to assess motor coordination, and are suggested to nicely reflect walking deficits in PD patients, since PD has been clinically characterized by increases in gait variability (Pistacchi et al., 2017). During the early stages of the disease, rodents experience minor reductions in stride length and walking speed. In the middle stages, gait abnormalities become more pronounced, with shuffling steps and reduced arm swing. By the advanced stages, severe gait impairments are evident, often including episodes of freezing of gait, which severely impact mobility and quality of life (Di Biase et al., 2020).

There are two approaches developed for performing gait analysis; one is based on a manual application known as the footprint test or ink-test (Figure 3E), while the other makes use of an automated treadmill to measure stride length of the animal (Glajch et al., 2012). The manual gait analysis uses paint colors and paper for limb movement analysis, and animals are trained to walk on a narrow walkway often leading to their home cages or food rewards (Tillerson et al., 2002). The animal's stride length is determined by measuring the distance between the footprints examining both forelimb and hind paw strides (Tillerson et al., 2002). While the treadmill gait analysis uses an imaging apparatus and video cameras to measure animal's stride length (Tillerson et al., 2002). Animals are placed on a motorized treadmill often found in a plexiglass compartment; a camera is also placed underneath the treadmill used for visualizing paw contacts on the treadmill (Tillerson et al., 2002). Both applications of the test aim at not only analyzing the stride length, but also measuring all of the following gait features: variability between strides, area touched and possible overlap between hind and fore paw use (Brooks and Dunnett, 2009).

Gait dynamics have been assessed in MPTP treated mice, and results confirmed gait impairment due to MPTP treatment (Amende et al., 2005; Broom et al., 2017; Hampton and Amende, 2010; Wang et al., 2012b), where the mice's digital prints showed a significant decrease in their stride length, with an increase in stride frequency, in addition to having significantly shorter stride durations (Amende et al., 2005). Moreover, a recent study also confirmed shuffling behaviors resembling gait symptoms of advanced PD, associated with the loss of locomotor agility in MPTP treated mice (Geldenhuys et al., 2015). Similarly, studies of the 6-OHDA PD animal model reported abnormalities in gait dynamics in rat models of PD, where data indicated significant alterations in stride length, paw print position, print length, and print area and various other gait parameters all confirming the reliability of the approach for the detection of motor deficiencies in 6-OHDA lesioned rat models of PD (Boix et al., 2018). These data are concurrent with previous work confirming the utility of gait analysis in examining motor deficits induced by 6-OHDA (Zhou et al., 2015; Baldwin et al., 2017) (Table 1). Rotenone-induced locomotor deficits were also assessed with the application of gait dynamics testing, using the footprint test, data confirmed impaired motor coordination in rotenone treated rats (Madiha et al., 2017; Wen et al., 2011). Paraquat- and maneb-induced motor deficits were not as extensively examined using gait analysis, however one study did report impairments of gait performance in mice treated with both paraquat and maneb, with mice exhibiting shorter stride length and wider stride distance compared to their corresponding controls (Hou et al., 2018) (Table 1).

Observations of abnormalities in gait dynamics were not detected in Thy1 α-syn mice, with studies showing modest effects of the different gait parameters in this transgenic mouse model of PD (Fleming et al., 2006, 2004a). While footprint analysis of transgenic mice expressing mutant A53T human α-syn revealed significant reductions in the animal's step and stride length (Gispert et al., 2003), which was also confirmed in other studies showing motor disability, and gait asymmetry in A53T mice (Paumier et al., 2013; Tatenhorst et al., 2016; Lin et al., 2012; Hamill et al., 2012). This was also consistent in mice with the A30P mutation, with gait analysis showing significantly reduced stride length (Plaas et al., 2008; Mendritzki et al., 2010) (Table 1).

Similar to previous studies in transgenic rodent models of PD, not enough motor tests have been used in other transgenic PD models. One study reports no differences in footprint analysis between aged WT and Parkin-deficient mice (Perez and Palmiter, 2005) consistent with absence of significant motor deficits in this mouse model (Perez and Palmiter, 2005). While another study confirms gait alterations in the hind paws accompanied with an increase in the hind paw contact of Pink1-deficient mice (Glasl et al., 2012). These gait abnormalities were also reported in one study of DJ-1-deficient mice, with data showing significant variations in the animal's postural gait associated with shortened stride length and alterations in the mice's hind base displacement (Chandran et al., 2008). These behavioral examinations using the gait test were further confirmed in another study of DJ-1 deficient mice (Lev et al., 2015). Numerous studies using rodent transgenic models overexpressing WT or pathogenic variants of LRRK2 have reported conflicting data in the gait analysis when evaluating rodent behavior symptoms as reviewed in Seegobin et al. (2020) (Table 1).

#### 2.1.7 Rotarod test

The rotarod test is one of the oldest and best-defined methods to assess behavioral deficits in rodent models of PD, and this test was first described by Dunham and Miya in 1957 (Dunham and Miya, 1957). In the early stages of PD, rodents demonstrate a slight reduction in the time spent on the rod, reflecting initial motor impairments. As the disease progresses to the middle stages, a noticeable decrease in performance is observed, with animals struggling to maintain their balance for extended periods. In the advanced stages, there is a significant reduction in the time spent on the rod, often accompanied by frequent falls, mirroring the severe motor dysfunction seen in advanced PD in humans (Dunham and Miya, 1957).

The rotarod is an apparatus that consists of a circular rod that is around 3–7 cm in width, and the rod is placed to rotate at speeds that can be either constant or increasing depending on the type of analysis conducted (Shiotsuki et al., 2010; Brooks and Dunnett, 2009) (Figure 3F). The objective of the test is to investigate the balance and coordination of the animal (Shiotsuki et al., 2010; Brooks and Dunnett, 2009). The rodent is placed on the rotating rod where it is supposed to balance to try and remain on the rod avoiding falling onto a platform that is placed around 30 cm below the rod, the animal's latency to fall is recorded as the endpoint measure (Deacon, 2013). The test is widely used since it is rather simple to set up, and not much training is required for its application (Shiotsuki et al., 2010; Deacon, 2013).

The method used for the rotarod test is based on the training the animal receives with the objective of staying on the rod when the rod is subjected to either constant or increasing speed rotations (Deacon, 2013). In the constant rotation, rodents that repeatedly fall are placed back on the rotarod until they can stay on for at least 2 min (Carter et al., 2001; Brooks and Dunnett, 2009). Trained animals are then tested on a gradually accelerating rotarod, and the rotation speed begins at 5 rpm and is usually accelerated by 0.2 rpm per second (Carter et al., 2001; Brooks and Dunnett, 2009). While, for the constant speed rotarod test, mice are trained at an intermediate speed for 1 min and the latency to fall is recorded, with several trials conducted over several days (Carter et al., 2001; Brooks and Dunnett, 2009).

Deficiencies in the balance of animals and changes in their performance on the rotarod have been quite evident in lesioned and transgenic rodent models of PD. Interestingly, rotarod deficits have been classified as a “nondopamine dependent” motor behavior since as several studies have reported, they tend to appear preceding any observed dopaminergic loss (Magen and Chesselet, 2010; Campos et al., 2013).

A study examining rod performance in MPTP-treated-mouse models of PD, report that mice injected with MPTP show an evident decrease in performance on the rotarod compared to their controls, and the analysis were based on logging the number of falls at the end of the experiment in addition to the total time mice spend on the rotarod (Rozas et al., 1998). Consistently another study also reported that mice's rotarod performance shows significant reductions only 45 days post-MPTP lesions, using assessments based on the time spent on the rotarod and the speed survival of the animals (Ayton et al., 2013). This study also presented data that showed MPTP-lesioned mice stayed on the rotarod for shorter periods of time after receiving levodopa (L-Dopa) when compared to non-lesioned/L-Dopa supplemented mice, suggesting that an increase in dopamine levels with L-Dopa can indeed induce impaired rotarod performance in these animals (Ayton et al., 2013).

The rotarod test was also used for the assessment of motor deficits in the 6-OHDA rat model (Monville et al., 2006; Haddadi et al., 2014). Results from these studies also indicated lesioned 6-OHDA rats had higher latency to fall compared to normal rats, and this was evident both when using fixed-speed and the accelerating rotarod applications (Monville et al., 2006). These results were also confirmed in multiple other studies detecting significant motor impairment in 6-OHDA rodents using the rotarod test (Rozas et al., 1997; Rozas and Labandeira Garcia, 1997; Whishaw et al., 2003) (Table 1).

Furthermore, although not much work was done on paraquat models of PD, one study showed that when using the accelerating rotarod test, rats in the paraquat models displayed significant reductions in the latency to fall depicting deficiency in overall motor impairment (Campos et al., 2013). Similarly, paraquat + maneb treatment in rats induced deficiencies in motor coordination when using the rotarod test, with data showing that paraquat + maneb treatment significantly affects the time spent by the animals on the rotating bar (Tinakoua et al., 2015). One study evaluated motor behavior on rotenone-induced PD model, and the study reports a significant decrease in motor performance and coordination in their model (Magdy et al., 2022).

Moreover, deficiencies in motor function when applying the rotarod test were also examined in transgenic models of PD. In α-syn-overexpressing models, not many studies confirmed neuronal loss associated with motor dysfunction, one study showed that when using both human and mouse α-syn, mice did not show any motor deficits at 6 months after inoculation (Masuda-Suzukake et al., 2013). These results are concurrent with other studies, confirming that mice overexpressing α-syn do not show motor alterations on the rotarod until later stages of their development (Masliah et al., 2000; Al-Wandi et al., 2010). While transgenic mice expressing mutant A53T human α-syn show significant motor impairment on the rotarod test (Gispert et al., 2003), with several studies confirming that A53T mice do indeed develop changes in locomotor activity, exhibiting enhanced rotarod latency that declined with the age of the animal (Paumier et al., 2013). Initially, the studies reported motor improvement in A53T mice at their early or young ages, and this motor progression does suddenly decrease at 12 months of age (Paumier et al., 2013; Graham and Sidhu, 2010; Oaks et al., 2013). While these observations of age-dependent motor deficiencies, were not reflected in the A30P α-syn mouse model as observed using motor tests such as the rotarod (Plaas et al., 2008; Yan et al., 2017). Moreover, no motor distinctions using the rotarod test were observed for Parkin (Goldberg et al., 2003; Zhu et al., 2007), DJ-1 (Dave et al., 2014; Chen et al., 2005), PINK1 (Gispert et al., 2009; Zhou et al., 2007) and LRRK2 (Bichler et al., 2013) transgenic animal models of PD, for all these models exhibited similar latencies for remaining on the rotating rod when compared to their corresponding controls (Table 1).

### 2.2 L-DOPA induced abnormal involuntary movements (AIMs) test

The AIMs test has been used to reflect and measure dyskinesia, and catalepsy in rodent models of PD after administration of drugs like L-DOPA (Cenci et al., 1998). The AIMs test has been described in depth by Cenci et al. (1998), and is classified into different subtypes based on the type of measurement of the behavioral abnormality. These subtypes include measurements of: (i) locomotive dyskinesia also known as controversive rotational response characterized by the increase in locomotion, which is dependent on the contralateral side of treatment, (ii) contralateral twisted posturing of both the neck and upper body, (iii) orolingual dyskinesia, characterized by stereotyped jaw movements associated with contralateral tongue protrusion, and (iv) forelimb dyskinesia which is characterized by repetitive rhythmic jerks of the contralateral forelimb, also described by grabbing movements of the contralateral paw (Cenci et al., 1998; Lee et al., 2000). Scores are used to assess the scale of the behavioral abnormality; and the animal is thus scored on a scale from 0 to 4 (0, absent; 1, occasional; 2, frequent; 3, continuous; 4, continuous and severe, not interruptible by external stimulations) (Lundblad et al., 2002).

Observations are recorded based on the subtype of the test and scores are given for rodents placed in a transparent, open environment such as the glass cylinders utilized in the cylinder test, and the monitoring period ranges between 1 and 2 min during an assigned time block of 20–120 min (Park et al., 2016). The AIMs score given, corresponds to the sum of the individual scores for each subtype examined (Cenci et al., 1998; Park et al., 2016).

Many studies make use of the AIMs test to best reflect the clinical relevance of the PD models of akinesia and dyskinesia. In the MPTP model, efforts have been put toward the proper detection of motor abnormalities, but some studies were unable to detect impairments using AIMs scoring in MPTP-treated control mice (Rommelfanger et al., 2007). While more recent studies did show a substantial effect on aged mice treated with MPTP after L-DOPA treatment, showing physical signs of movement disability that is characterized by akinesia, rigidity of the hind limbs and resting tremor of the whole body (Gupta et al., 1986). Moreover, assessment of hindlimb clasping behavior in MPTP/L-DOPA treated mice also confirmed presentation of dyskinesia symptoms in this model (Lazzara et al., 2015). AIMs was also examined in 6-OHDA-lesioned rat models of PD, with an observed increase of dyskinesia in this model (Wan et al., 2017). The reported effect have been suggested to only show significant effects after chronic treatment with L-DOPA (Aristieta et al., 2012; Cenci and Lundblad, 2007; Issy et al., 2015; Andersson et al., 1999), with observed movements resembling dyskinesia in PD (Picconi et al., 2003). As reported by several studies, the effect of dyskinesia symptoms observed are predominantly established on the side of the body contralateral to the lesion (Picconi et al., 2003) (Table 1).

Moreover, studies have also focused on assessing rotational behavior when rodents are exposed to psychotropic compounds such as amphetamine and apomorphine. Amphetamine mode of action is based on its capacity to bind to the dopamine transporter, inducing the reversal of the protein dopamine allowing for cytosolic dopamine release and consequently causing what is referred to as dose-dependent motor activation (Kuhr et al., 1985; Sulzer et al., 1995). These drugs have been examined in models of 6-OHDA following chronic administration of different L-DOPA doses, allowing for greater dopamine release in the intact striatum side compared to the lesioned side, and this induction was reported to cause an asymmetric motor activation of the right and left sides of the body, resulting in an ipsilateral rotational behavior (Schwarting and Huston, 1996; Putterman et al., 2007; Tronci et al., 2012; Iancu et al., 2005).

As for the drug apomorphine, studies have shown that it induces a weaker correlation with nigral cell loss and rotational behavior than what has been reported for amphetamine in rodent models of PD. Apomorphine, similar to amphetamine, is a dopamine receptor agonist suggested to cause contralateral turning by stimulating dopamine receptors (Creese et al., 1977). The difference between the two drugs (apomorphine and amphetamine), is that apomorphine receptor upregulation does not occur until a high percentage of dopamine afferents are actually lost (Creese et al., 1977). One of the most examined rotational behaviors induced by apomorphine induction is in rodents lesioned by unilateral microinjection of 6-OHDA. Work on rats injected with 6-OHDA and further challenged with apomorphine showed an induction in contralateral rotation, which has been suggested to be due to the sensitivity in the 6-OHDA lesioned hemisphere (Ungerstedt, 1971; Creese et al., 1977; Silverman and Ho, 1981; Waddington et al., 1979). This behavioral characterization has also been examined in 6-OHDA lesioned mice, but data reported showed inconsistencies in the performance of apomorphine-induced rotation (Iancu et al., 2005). While no work has examined the use of AIMs testing in the other toxin-induced models of PD such as rotenone, paraquat and paraquat + maneb. Similarly, no AIMs test has been reported for the transgenic and genetic models of PD (Table 1).

## 3 Non-motor behavioral tests

### 3.1 Depression-like behavior

Cognitive decline is one of the most frequent and debilitating non-motor symptoms of PD (Gonzalez-Latapi et al., 2021). This decline is frequently accompanied by a variety of behavioral alterations such as depression, anxiety, and cognitive impairment (Degirmenci et al., 2023).

A large number of studies examining PD-like behavior in animals have indicated that many of the PD models present with depressive-like behavior as reviewed in Fontoura et al. (2017).

In order to best assess behavioral alterations evaluated in toxin-induced or genetic models of PD, animal are usually submitted to tests such as (i) forced swimming test (FST) and (ii) sucrose-consumption test (SCT) which are both essentially designed to evaluate depressive behavior in rats and mice (Fontoura et al., 2017). Data from these studies have proven that behavioral tests used to infer depressed-like behavior in rodent models of PD can help provide useful data when and if the appropriate animals and proper methods are implemented.

#### 3.1.1 Forced swimming test (FST)

The FST is one of the most used tests to evaluate depression-like behavior in mice and rats. In early stages of PD, rodents exhibit a slight increase in immobility time during the FST, indicating emerging depression-like behavior. As the disease progresses to middle stages, a noticeable increase in immobility is observed, paralleling worsening depressive symptoms. By the advanced stages, animals show significant immobility, reflecting severe depressive-like behavior, which often accompanies advanced PD (Porsolt et al., 1978).

**Figure 4:**
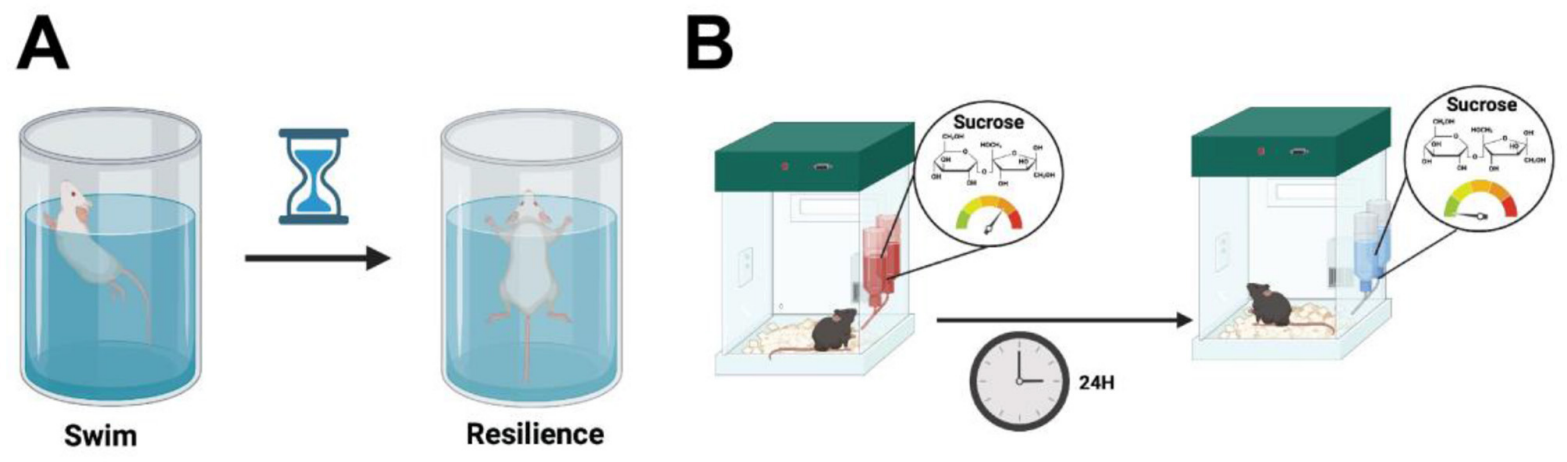
**(A)** In the forced swim test, rodents are placed in a vertical plexiglass cylinder filled with water. After a 24 h training period, a 5 min test session is conducted to measure despair, quantified by the total duration of immobility, where the animal ceases escape attempts. **(B)** In the sucrose preference test, animals are placed in home cages with two pre-weighed water bottles for 24 h for habituation. The water is then replaced with a low percentage sucrose solution and after another 24 h, the bottle positions are swapped. Total intake of water and sucrose is measured to compare the percentage of sucrose consumption between groups. Created using BioRender.com source.

The model was first described by Porsolt et al. (1978), and the test is based on the observations collected when rodents are forced to swim in a restricted, inescapable space, where eventually the animal becomes immobile, ceasing their attempts to escape (Porsolt et al., 1978). This behavioral assay consists of placing the animal in a vertical plexiglass cylinder filled with water, and after 24 h of training, a short session of around 5 min is carried out. The rodent's despair is analyzed by measuring the total duration of immobility during the test (Porsolt et al., 1978) (Figure 4A).

Several studies report variabilities with this test among the different PD models. Rats exposed to MPTP, 6-OHDA, rotenone and paraquat show significant reductions in their swimming time using the FST test, while only the 6-OHDA neurotoxin model presented with a clear increase in immobility time during the test (Tadaiesky et al., 2008; Santiago et al., 2010; Bonito-Oliva et al., 2014). Moreover, the total immobility time was affected in the paraquat-induced rat model, but not in 6-OHDA-treated rats as reported by a more recent study (Campos et al., 2013). These reports describe the discrepancies in these models in different studies examining depression-like behavior in PD animal models (Table 2).

**Table 2:** Summary of non-motor tests evaluated in Parkinson's disease models.

	**Non-motor cognitive tests**								
		**Depression**		**Anxiety**	**Memory**				**Reference:**
		**SCT**	**FST**	**OFT**	**EPM**	**MWMT**	**ORT**	**Y-MT**	
Neurotoxin	6-OHDA	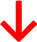 ^1^	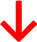 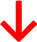 ^2^	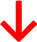 ^3^	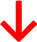 ^4^	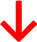 ^5^	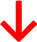 ^6^	?	1. Tadaiesky et al., 2008; Santiago et al., 2010; Campos et al., 2013; Carvalho et al., 2013; Silva et al., 2016. 2. Tadaiesky et al., 2008; Santiago et al., 2010; Bonito-Oliva et al., 2014; Campos et al., 2013. 3. Bonito-Oliva et al., 2014; Vieira et al., 2019 4. Campos et al., 2013; Silva et al., 2016. 5. Ferro et al., 2005; Tadaiesky et al., 2008; Campos et al., 2013 6. Goes et al., 2014.
	MPTP	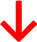 ^1^	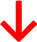 ^2^	?	?	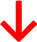 ^3^	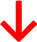 ^4^	?	1. Santiago et al., 2010 2. Santiago et al., 2010 4. Ferro et al., 2005; Prediger et al., 2010 5. Ho et al., 2014 Yabuki et al., 2014
	Rotenone	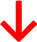 ^1^	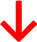 ^2^	?	?	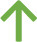 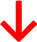 ^3^	?	?	1. Santiago et al., 2010 2. Santiago et al., 2010 3. Jia et al., 2014
	Paraquat	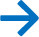 ^1^	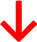 ^2^	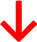 ^3^	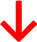 ^4^	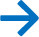 ^5^	?	?	1. Campos et al., 2013 2. Santiago et al., 2010; Campos et al., 2013 3. Litteljohn et al., 2009 4. Campos et al., 2013; 5. Campos et al., 2013
Genetic model	Thy1 α-syn	?	?	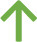 ^1^	?	?	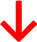 ^2^	?	1. Wang et al., 2012a 2. Chesselet et al., 2012
	α-syn A53T	?	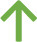 ^1^	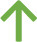 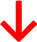 ^2^	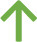 ^3^	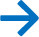 ^4^	?	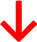 ^5^	1. Oaks et al., 2013 2. Graham and Sidhu, 2010; Paumier et al., 2013 3. George et al., 2008; Graham and Sidhu, 2010 4. Liu et al., 2018 5. Paumier et al., 2013
	α-syn A30P	?	?	?	?	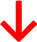 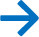 ^1^	?	?	1. Freichel et al., 2007
	Parkin	?	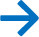 ^1^	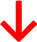 ^2^	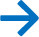 ^3^	?	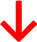 ^4^	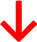 ^5^	1. Perez and Palmiter, 2005; Rial et al., 2014 2. Zhu et al., 2007 3. Perez and Palmiter, 2005; Rial et al., 2014 4. Perez and Palmiter, 2005; Rial et al., 2014 5. Rial et al., 2014
	Pink1	?	?	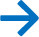 ^1^	?	?	?	?	1. Gispert et al., 2009
	DJ-1	?	?	?	?	?	?	?	
	LRRK2	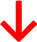 ^1^	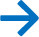 ^2^	?	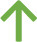 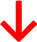 ^3^	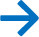 ^4^	?	?	1. Lim et al., 2018 2. Lim et al., 2018 3. Lim et al., 2018. 4. Shaikh et al., 2015

Depression tests: 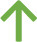
= less depressive behavior, 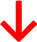
= more depressive behavior, 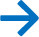
= no effect on depressive behavior. Anxiety tests: 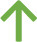
= less anxiety, 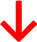
= more anxiety, 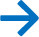
= no effect on anxiety. Memory tests: 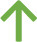
= better performance, 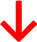
= worse performance 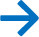
= no significant change. ? = no reported studies.

Among the models overexpressing human α-syn, Oaks et al. (2013) reported reductions in the depressive-like behavior in aged A53T mice (Oaks et al., 2013), while young (2-month old) A53T mice presented with similar immobility time to their corresponding controls during the FST (Oaks et al., 2013). Moreover, rat models overexpressing human WT α-syn did not result in depressive-like behavior (Wan et al., 2016). Similar results were also previously observed in other studies examining depressive behaviors in α-syn rat models (Campos et al., 2013; Caudal et al., 2015). Other models such as parkin-deficient mice displayed normal performance in FST, showing a behavioral profile similar to control mice (Perez and Palmiter, 2005; Rial et al., 2014) (Table 2).

Recently, Lim et al. (2018) have examined both anxiety and depression-like behavior in transgenic male and female mice expressing human mutant LRRK2 (Lim et al., 2018). Their study reports longer immobility time during the FST in middle-aged (43–52 weeks) LRRK2 animals as compared with the non-transgenic controls (Lim et al., 2018). Older mice were excluded from this test because of the impaired motor function observed in the transgenic group (Lim et al., 2018) (Table 2).

#### 3.1.2 Sucrose-consumption test (SCT)

The SCT, also referred to as sucrose preference test, is frequently used to evaluate hedonic anhedonia, defined as the decrease in interest in response to formerly rewarding stimulias observed in rodents (Papp et al., 1991; Wang et al., 2009). In the early stages of PD, rodents exhibit a slight reduction in sucrose preference, suggesting the onset of anhedonia. As the disease progresses to the middle stages, there is a noticeable decrease in sucrose consumption, reflecting a more pronounced loss of interest in rewarding stimuli. By the advanced stages, a significant reduction in sucrose consumption is observed, indicating severe anhedonia, a hallmark of advanced PD-related depressive-like behavior (Berrio and Kalliokoski, 2023).

In the SCT test, animals are individually placed in home cages with two pre-weighed bottles of water placed on the extreme sides of the cage (Slattery et al., 2007; Santiago et al., 2010), and after a period of 24 h, allocated for the animal's habituation, water consumption from both bottles is measured (Slattery et al., 2007; Santiago et al., 2010) (Figure 4B). The content of the bottles is then replaced by a low percentage sucrose solution usually ranging between 0.8 and 3% (Slattery et al., 2007; Santiago et al., 2010). Twenty-four hours post water content change, the location of the bottles is reversed, and the total intake is evaluated 24 h later for both water and sucrose consumption (Slattery et al., 2007; Santiago et al., 2010). The behavioral analysis is then based on comparing the percentage of sucrose intake by the different groups (Slattery et al., 2007; Santiago et al., 2010).

Several studies reported a decrease of sucrose consumption in male rats exposed to 6-OHDA, MPTP and rotenone, revealing depressive-like behavior in most of the neurotoxin models of PD (Campos et al., 2013; Santiago et al., 2010; Tadaiesky et al., 2008; Silva et al., 2016; Carvalho et al., 2013). While, young adult male rats exposed to paraquat did not show to exhibit anhedonia-like behavior when observed using SCT (Campos et al., 2013) (Table 2).

Not many studies have evaluated depression-like behavior in PD genetic models using SCT. Studies reported a depressive-like behavior in rats expressing human WT α-syn both in FST (as previously described) and in SCT (Caudal et al., 2015). Similarly, both middle-aged (43–52 weeks) and old (65–83 weeks) LRRK2 mice also showed to exhibit significant reductions in their sucrose preference confirming depression-like behavior in this PD model (Lim et al., 2018) (Table 2).

### 3.2 Anxiety-like behavior

Similar to depression, anxiety is considered to be one of the major non-motor symptoms observed in PD patients (Taylor et al., 2010). Interestingly anxiety-like behaviors have been successfully measured in several of the animal models of PD, and numerous methods have been described and used to study anxiety in PD rodents. The most used methods are the (i) open field test, and the (ii) elevated plus maze (EPM).

#### 3.2.1 Open field test

As previously described, the open field test is a largely used behavioral assay used to assess novel environment exploration and general locomotor activity (Santiago et al., 2010; Seibenhener and Wooten, 2015; Tatem et al., 2014) (Figure 2A). This test can also provide a screening evaluation of anxiety-like behavior in rodents (Prut and Belzung, 2003). During the first few minutes in the open field, animals tend to mainly explore the peripheral zone of the open field (Simon et al., 1994). The degree of thigmotaxis (tendency to remain close to the walls) can be measured as an index of anxiety in experimental mice and rats, in addition to measuring the total distance traveled and the total immobility time throughout the duration of the test (Simon et al., 1994).

Some studies have reported anxiety-like phenotypes in neurotoxin-induced models of PD when using the open field test. 6-OHDA lesioned mice, for example, were reported to spend less time in the center of the open field showing increased thigmotaxis when compared to their controls (Bonito-Oliva et al., 2014; Vieira et al., 2019). Similarly, paraquat treatment also showed to progressively affect the open field exploration of mice 1–3 weeks post-treatment, associated with reductions in the number of entries and exploration of the animal in the central zone of the open field (Litteljohn et al., 2009) (Table 2).

Among the genetic models of PD, an increased thigmotaxic behavior was reported for parkin-deficient mice, described with reductions in the animal's time spent in the center of the field (Zhu et al., 2007). While in Pink1 transgenic mice, it was reported that the time the animal takes to explore the peripheral and the central zone of the open field does not appear to differ to the control mice (Gispert et al., 2009) (Table 2).

Open field test results revealed not only an increase in activity, but also reduced anxiety-like behavior in transgenic A53T mouse models (Graham and Sidhu, 2010; Paumier et al., 2013). These studies show that A53T mice seemed to develop an age-related anxiolytic-like phenotype (Graham and Sidhu, 2010; Paumier et al., 2013). In fact, 12-month-old A53T mice showed to spend more time in the center as compared to either WT or younger A53T mice (Graham and Sidhu, 2010; Paumier et al., 2013). Similarly, another study showed that mice overexpressing the human WT α-syn (Thy1-α-syn) tend to have more center entries and exploration time when compared to WT mice (Wang et al., 2012a) (Table 2).

#### 3.2.2 Elevated plus maze (EPM)

The EPM test evaluates anxiety-related responses in rodents by measuring their preference for closed vs. open arms. Increased avoidance of open arms is indicative of heightened anxiety levels. In the early stages of PD, rodents show a slight increase in the time spent in the closed arms, reflecting emerging anxiety-like behavior. During the middle stages, this behavior becomes more pronounced, with a noticeable preference for the closed arms as anxiety symptoms intensify. By the advanced stages, rodents exhibit significant avoidance of the open arms, indicating severe anxiety, which is often observed alongside motor and other non-motor deficits in advanced PD (Pellow et al., 1985).

**Figure 5:**
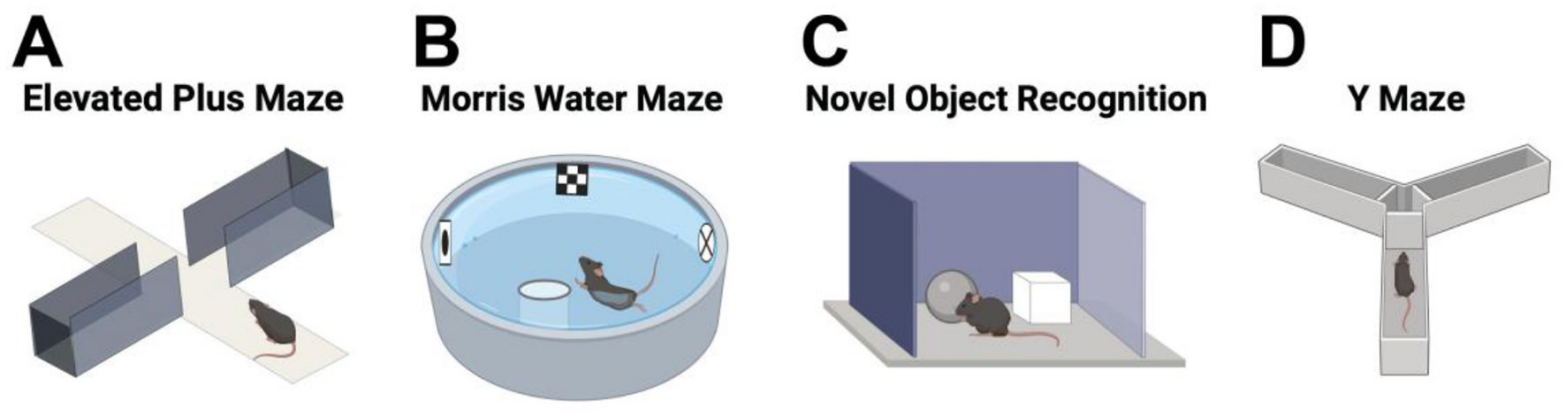
**(A)** The Elevated Plus Maze test uses a four-arm device to assess anxiety in rodents. The apparatus has alternating open and enclosed arms forming a plus shape. Time spent in the open arms vs. the enclosed arms indicates anxiety levels. **(B)** In the Morris Water Maze, a circular pool is filled with opaque water and a hidden platform just below the surface. Rodents are placed in the pool, and the test measures their ability to use external spatial cues to find the hidden platform. Performance is analyzed by recording the time taken to reach the platform and escape from the water. **(C)** The Object Recognition Test assesses a rodent's ability to recognize novelty. Initially, the rodent explores two identical objects (acquisition trial). After a delay, one object is replaced with a novel one for the retention trial. Performance is analyzed by comparing the time spent exploring the novel object to the familiar one, using a recognition/discrimination index. **(D)** The Y-Maze test examines the animal's preference for exploring a new arm of the maze. The Y-shaped maze consists of three opaque arms set at 120° angles from each other. The rodent is placed at the center and allowed to freely explore all three arms. Over time, the animal is expected to show a preference for entering the less recently visited arm. Created using BioRender.com source.

The EPM is a four-arms device commonly used to assess anxiety response in rodents (Pellow et al., 1985). The apparatus consists of alternating open and enclosed arms with a common central platform that is arranged to form a plus-like shape (Handley and Mithani, 1984) (Figure 5A). This device was modified from the Y-shape apparatus initially described by Montgomery (1955), with an elevated open alley (associated with strong approach-avoidance conflict) and an enclosed alley. The time spent in the open arms compared to the time spend in the closed arms is used as an anxiety indicator (Pellow et al., 1985; Handley and Mithani, 1984). In fact, this task is based on rodents' proclivity toward dark and enclosed spaces known as (approach) and an unconditioned fear of heights/open spaces known as (avoidance) (Lezak et al., 2017). Thus, a decrease in open-arm activity (time and/or entries) are the criteria used to reflect anxiety-like behavior in animal models of PD (Lim et al., 2018).

Different studies have observed what is referred to as the “anxious phenotype” in several of the neurotoxin-induced models of PD as detected using the EPM task. Paraquat and 6-OHDA lesioned rats showed significant reductions in the time spent in the open arms of the EPM compared to control animals, showing anxiety-like behavior (Silva et al., 2016; Campos et al., 2013). Silva et al. (2016) also noted a significant decrease in the number of arm entries in the 6-OHDA-lesioned animal group (Silva et al., 2016). The study by Campos et al. (2013), also described a tendency for rats overexpressing human WT α-syn (driven by the U6 promoter), to spend less time in the open arms (Campos et al., 2013). However, another study examining rats overexpressing human WT α-syn (driven by tryptophan hydroxylase promoter), reported reductions in the anxiety-like behavior of these animals (Wan et al., 2016). This group of rats also displayed higher open arm entries and longer times spent both in the open arms and the central area compared to control subjects (Wan et al., 2016). On the other hand, A53T transgenic mice exhibited a reduced anxiety-like behavior on the EPM (Graham and Sidhu, 2010; George et al., 2008). In fact, A53T transgenic mice showed to spend significantly longer time and greater number of entries in the open arms than their corresponding controls (Graham and Sidhu, 2010; George et al., 2008) (Table 2).

Moreover, other genetic models of PD present anxiety-like behavior. Middle-aged (43–52 weeks) and old (65–83 weeks) LRRK2 transgenic mice exhibited both significant reductions in the number of entries in the open arms and a significant increase in closed-arms entries when compared to the controls (Lim et al., 2018). On the contrary, Parkin-deficient mice performance in the EPM task was strikingly similar to control animals, indicating that this transgenic model does not present with anxiety-like behavior (Perez and Palmiter, 2005; Rial et al., 2014) (Table 2).

### 3.3 Cognitive impairment

Cognitive impairments associated with PD have been linked with deficits in attention (Ballard et al., 2002), and working memory (Kehagia et al., 2010) affecting both spatial (Levin, 1990) and nonspatial working memory (Matison et al., 1982), in addition to visual perception (Levin, 1990; Lee et al., 1998) and object recognition (Laatu et al., 2004). Now that cognitive impairment in PD patients has become a highly relevant issue, preclinical studies on animal models of PD have presented data that show that PD animal models have spatial memory, attention, and learning deficits related to the disease. Some of the most widely used tests examining cognitive impairment in animal models of PD include the (i) Morris water maze test, (ii) object recognition test, and the (iii) Y-maze task.

#### 3.3.1 Morris water maze test (MWMT)

Working and spatial memory can be assessed using the Morris water maze, which involves a circular pool filled with opaque water and a hidden platform placed just below the surface (Morris et al., 1982). Rodents are placed in the pool, and the test then measures the capacity of the animal to make use of external spatial cues to locate the platform placed in the pool (Morris et al., 1982). The animal performance during the test is analyzed by measuring the time the animal takes to reach the platform, and the time it takes to escape from the water; longer escape times can indicate memory impairment (Morris et al., 1982) (Figure 5B). In the context of PD progression, rodents show a slight increase in the time it takes to find the platform, reflecting initial cognitive deficits. As the disease progresses to the middle stages, more noticeable impairments in learning and memory become apparent, with rodents requiring longer times or exhibiting inconsistent navigation strategies. By the advanced stages, severe deficits are evident, with rodents often unable to locate the platform, indicating significant deterioration in spatial memory and cognitive function (Morris et al., 1982). Different results have been obtained for neurotoxin-induced models of PD. The performance of this task was not affected by paraquat exposure in young male rats (Campos et al., 2013). On the contrary, rats lesioned by 6-OHDA showed memory impairment when tested using the Morris water maze test (Tadaiesky et al., 2008; Campos et al., 2013; Ferro et al., 2005). These studies showed that there were significant daily improvements in the performance of control animals in finding the platform, which was not observed for the 6-OHDA group (Tadaiesky et al., 2008; Campos et al., 2013; Ferro et al., 2005). A similar effect was reported in MPTP-lesioned rats (Ferro et al., 2005) and mice (Prediger et al., 2010). Furthermore, a study by Jia et al. (2014) reported that spatial memory was enhanced in mice treated with rotenone for 3 months but reduced in 1-month-treated animals (Jia et al., 2014).

Among the PD genetic models, no significant differences in working, spatial learning and spatial memory were detected between rats expressing human mutated LRRK2 and WT controls when evaluated with the Morris water maze test, which was examined by analyzing the animal's escape latency time (Shaikh et al., 2015).

Additionally, most of the α-syn transgenic models do not seem to show memory impairment in the Morris water maze test. In fact, the performance of rats overexpressing human WT α-syn was not different from their corresponding controls (Campos et al., 2013; Wan et al., 2016). Likewise, no significant differences in the escape latency were found in mice expressing mutated A53T α-syn (Liu et al., 2018). On the other hand, A30P mice displayed an age-dependent cognitive decline, where it was reported that at 4 months of age, results from the test did not differ between the transgenic and the control groups (Freichel et al., 2007). However, the performance of 12-month-old A30P mice showed significant impairment when compared to their corresponding controls (Freichel et al., 2007) (Table 2).

#### 3.3.2 Object recognition test (ORT)

The object recognition test (ORT) is based both on the rodent ability to discriminate familiarity and on their natural preference for novelty (Ennaceur and Delacour, 1988). This behavioral task consists of presenting the animal with two identical copies of one object (generally in an open box) (Ennaceur and Delacour, 1988). The animal is then allowed to freely explore the box and the objects (acquisition trial) before being placed back in its home cage (Ennaceur and Delacour, 1988). After a given delay, one of the objects is replaced with a different object (novel object) while keeping the other object in the cage (familiar object), and the animal is once again placed in the open box for the retention trial (Ennaceur and Delacour, 1988) (Figure 5C). The test performance is analyzed by measuring the time spent exploring the novel object compared to the time spent exploring the familiar object, and the test read out is based on the recognition/discrimination index (Ennaceur and Delacour, 1988). Healthy rodents tend to spend more time exploring the novel object, indicating good memory function (Ennaceur and Delacour, 1988). In contrast, in the early stages of PD, rodents show a slight reduction in the time spent exploring novel objects, suggesting early cognitive deficits and subtle memory impairments. As the disease progresses to the middle stages, a more noticeable preference for familiar objects becomes apparent, indicating a decline in the ability to remember the novel object. By the advanced stages, severe memory impairments are observed, with rodents showing no preference for the novel object, indicating a significant loss of recognition memory and cognitive function (Ennaceur and Delacour, 1988). The difficulty of the task can be modified depending on the duration of the acquisition trial, the retention delay, and the degree of similarity between the objects (Barker and Warburton, 2011).

Different studies reported cognitive dysfunction both in neurotoxin-induced and genetic models of PD using the ORT. 6-OHDA lesioned mice exhibited lower recognition index as compared to the control vehicle-treated mice (Goes et al., 2014). Similarly, MPTP lesioned rats (Ho et al., 2014) and mice (Yabuki et al., 2014) were showen to spend less time exploring the novel object compared to non-lesioned animals (Yabuki et al., 2014; Ho et al., 2014).

Parkin-deficient mice failed to discriminate between familiar and novel objects during the test, and also showed to exhibit a lower recognition index when compared to the control mice, suggesting short-term spatial memory deficits in this transgenic model (Perez and Palmiter, 2005; Rial et al., 2014). Additionally, novel and spatial object recognition memories were also disrupted in mice expressing human WT α-syn under Thy-1 promoter (Chesselet et al., 2012) (Table 2).

#### 3.3.3 Y-maze task (Y-MT)

Similar to ORT, the Y-maze task is based on rodents' innate tendency to explore novelty (Wolf et al., 2016). The objective of the test is to examine the preference of the animal to explore a new arm of the maze (Wolf et al., 2016). In the early stages of PD, rodents show a slight reduction in alternation percentage, suggesting subtle impairments in working memory. This initial decline reflects the early cognitive deficits associated with the disease. As PD progresses to the middle stages, noticeable impairments in working memory become evident, with rodents demonstrating a reduced ability to alternate between the arms of the maze. By the advanced stages, there is a significant reduction in alternation behavior, indicating severe memory deficits (Wolf et al., 2016).

The setup of the task is based on the use of a Y-shaped maze with three opaque arms usually placed at a 120° angle from each other (Miedel et al., 2017). A rodent is placed in the center of the maze and is allowed to freely explore the three arms, over the period of arm entries, the animal should eventually show tendency in its preference to enter a less recently visited arm (Miedel et al., 2017) (Figure 5D). Data acquisition is based on calculating the number of arm entries in order to assess the percentage of alternations made by the animal (Miedel et al., 2017). This test allows for the proper quantification of cognitive deficits in PD animal models (Miedel et al., 2017).

The Y-maze task has not been extensively used to evaluate cognitive impairment in neurotoxin-induced models of PD. However, a few studies reported poor task performance in some of the PD genetic models. Short-term spatial memory seems to be disrupted in Parkin mice, as reflected by studies examining spatial memory using ORT (Rial et al., 2014). Likewise, the Parkin transgenic model was shown to exhibit poor performance in the Y-maze task, indicated by a decrease in the time spent in the novel arm (Rial et al., 2014). Furthermore, a study by Paumier et al. (2013) reported an age-dependant spatial memory deficit in A53T mutated mice, where their data showed that 2-month old A53T mice tend to spend more time exploring and entering the novel arm, suggesting intact spatial memory (Paumier et al., 2013). However, at 6 and 12 months of age, A53T mice showed to spend less time in the novel arm compared to their corresponding controls, failing to discriminate between the familiar and novel arms (Paumier et al., 2013) (Table 2).

## 4 Conclusions

The diverse array of motor and non-motor tests utilized in PD research underscores the complexity of this neurodegenerative disorder and the multifaceted nature of its symptoms. From assessments targeting motor impairments, such as locomotor activity and induced rotational behavior, to those focusing on non-motor symptoms like depression, anxiety, and cognitive decline, these tests provide invaluable insights into the pathophysiology and progression of PD. Understanding the nuances of each test and its relevance to the aspects of PD being investigated is essential for accurately interpreting experimental data and drawing meaningful conclusions. By carefully matching test methodologies with the desired outcomes, researchers can enhance the validity and reproducibility of their findings, ultimately advancing our understanding of PD pathophysiology and improving the translational potential of preclinical research.

Importantly, although rodent models are the most commonly used in PD research, each one can help address specific aspects of the pathological mechanisms of the disease. On one hand, toxin-based models tackle the investigation of oxidative stress and selective DA neuronal vulnerability, making them optimal for studying neuroprotective approaches. However, they come with several limitations, including rapid and massive neurodegeneration that precludes the study of disease progression. Moreover, these models fail to exhibit the pathological accumulation of α-syn, a key feature of PD and related disorders. On the other hand, genetic models allow for the study of molecular mechanisms linked to familial forms of PD associated with specific mutations in different PD-related genes. These models are useful for studying protective approaches, identifying pharmacological targets, and understanding the implications of PD-related proteins in disease pathogenesis. However, they also have limitations, such as the absence of dopaminergic degeneration in several transgenic α-syn models, which only provide clues on synaptic dysfunction without clear neurodegeneration, representing early stages of the disease.

Clearly, the best model does not exist; rather, the optimal model can be chosen based on the scientific question, the disease mechanism under study, and the symptoms that the approach aims to improve. Fortunately, new technologies can further enhance existing animal models, particularly genetic-based PD models. For instance, optobiology now offers more spatiotemporal resolution in studying α-syn pathology in PD and related disorders. Recent optogenetic models, such as the Light-Inducible Protein Aggregation (LIPA) system, have emerged as powerful tools to dissect PD pathology with high temporal and spatial precision (Berard et al., 2022). This innovative technique has the potential to elucidate the dynamic processes of protein aggregation, clearance, and neurodegeneration in living systems, areas where conventional models often fall short.

Moreover, improvements in viral delivery systems are enhancing protein overexpression-based models. For instance, the development of AAV-PHP capsids allows for systemic injection of viral particles, enabling widespread overexpression of α-syn in the brain and selective DA neuronal loss associated with progressive motor impairment (Berard et al., 2023). Finally, gene-editing tools like CRISPR/Cas9 have revolutionized the development of PD models. These tools allow for precise genetic modifications to introduce or correct mutations associated with familial PD. For motor pathologies, CRISPR has been used to create knockout or knock-in models of PD-associated genes such as α-syn to model dopaminergic neuron degeneration and neuroinflammation (Luo et al., 2019; Qu et al., 2023). In non-motor pathologies, CRISPR has been used to investigate cognitive impairment, mood disorders, and autonomic dysfunction by manipulating genes related to synaptic plasticity, neurotransmitter systems, and circadian rhythms (Jiang et al., 2024; Ke et al., 2021).

In summary, the continuous development and refinement of PD models, combined with innovative technologies, are crucial for advancing our understanding of PD. These efforts will ultimately lead to more effective therapeutic strategies and improved outcomes for patients suffering from this debilitating disorder.
